# Alterations in Fibronectin Type III Domain Containing 1 Protein Gene Are Associated with Hypertension

**DOI:** 10.1371/journal.pone.0151399

**Published:** 2016-04-11

**Authors:** Alan Y. Deng, Cristina Chauvet, Annie Ménard

**Affiliations:** Research Centre, CRCHUM (Centre hospitalier de l’Université de Montréal), Department of Medicine, Université de Montréal, Montréal, Québec, Canada; Nazarbayev University, KAZAKHSTAN

## Abstract

Multiple quantitative trait loci (QTLs) for blood pressure (BP) have been detected in rat models of human polygenic hypertension. Great challenges confronting us include molecular identifications of individual QTLs. We first defined the chromosome region harboring *C1QTL1* to a segment of 1.9 megabases that carries 9 genes. Among them, we identified the gene encoding the fibronectin type III domain containing 1 protein (*Fndc1*)/activator of G protein signaling 8 (*Ags8*) to be the strongest candidate for *C1QTL1*, since numerous non-synonymous mutations are found. Moreover, the 5’ *Fndc1*/*Ags8* putative promoter contains numerous mutations that can account for its differential expression in kidneys and the heart, prominent organs in modulating BP, although the Fndc1/Ags8 protein was not detectable in these organs under our experimental conditions. This work has provided the premier evidence that *Fndc1*/*Ags8* is a novel and strongest candidate gene for *C1QTL1* without completely excluding other 8 genes in the *C1QTL1*-residing interval. If proven true by future *in vivo* function studies such as single-gene *Fndc1*/*Ags8* congenics, transgenesis or targeted-gene modifications, it might represent a part of the BP genetic architecture that operates in the upstream position distant from the end-phase physiology of BP control, since it activates a Gbetagamma component in a signaling pathway. Its functional role could validate the concept that a QTL in itself can influence BP ‘indirectly’ by regulating other genes downstream in a pathway. The elucidation of the mechanisms initiated by *Fndc*/*Ags8* variations will reveal novel insights into the BP modulation via a regulatory hierarchy.

## Introduction

Localization of quantitative trait loci (QTLs) has disclosed numerous chromosome regions as well as gene markers that are functionally associated with blood pressure (BP) in both humans [1] and animal models [2,3]. The genetic architecture of BP is composed of multiple QTLs as building blocks [4]. Their cumulative impact on the overall BP homeostasis has been revealed by experimental studies, and consists of limited modularized functional ‘blocks’ that fall into epistatic hierarchies [5]. In order to understand molecular mechanisms underlying the functionality of epistatic modules, we embarked on identifying gene variants causal to BP variations.

We previously defined several BP QTLs closely-linked on Chromosome (Chr) 1 in congenic strains cross-bred from hypertensive Dahl salt-sensitive (DSS) and Lewis rats [5]. They belong to one of the 2 epistatic modules from which QTLs take part in molding the overall BP [5]. In the present work, we aimed at identifying a likely causal gene candidate for such a QTL combining congenic resolution [6], a total genome sequencing and molecular analyses. We have identified potentially function-altering genetic variants in fibronectin type III domain containing 1 (*Fndc1*) that qualifies it to be a candidate, despite mouse knockout strains do not exist beyond embryonic cell lines [7].

## Methods

### Animals

**Fig 1:**
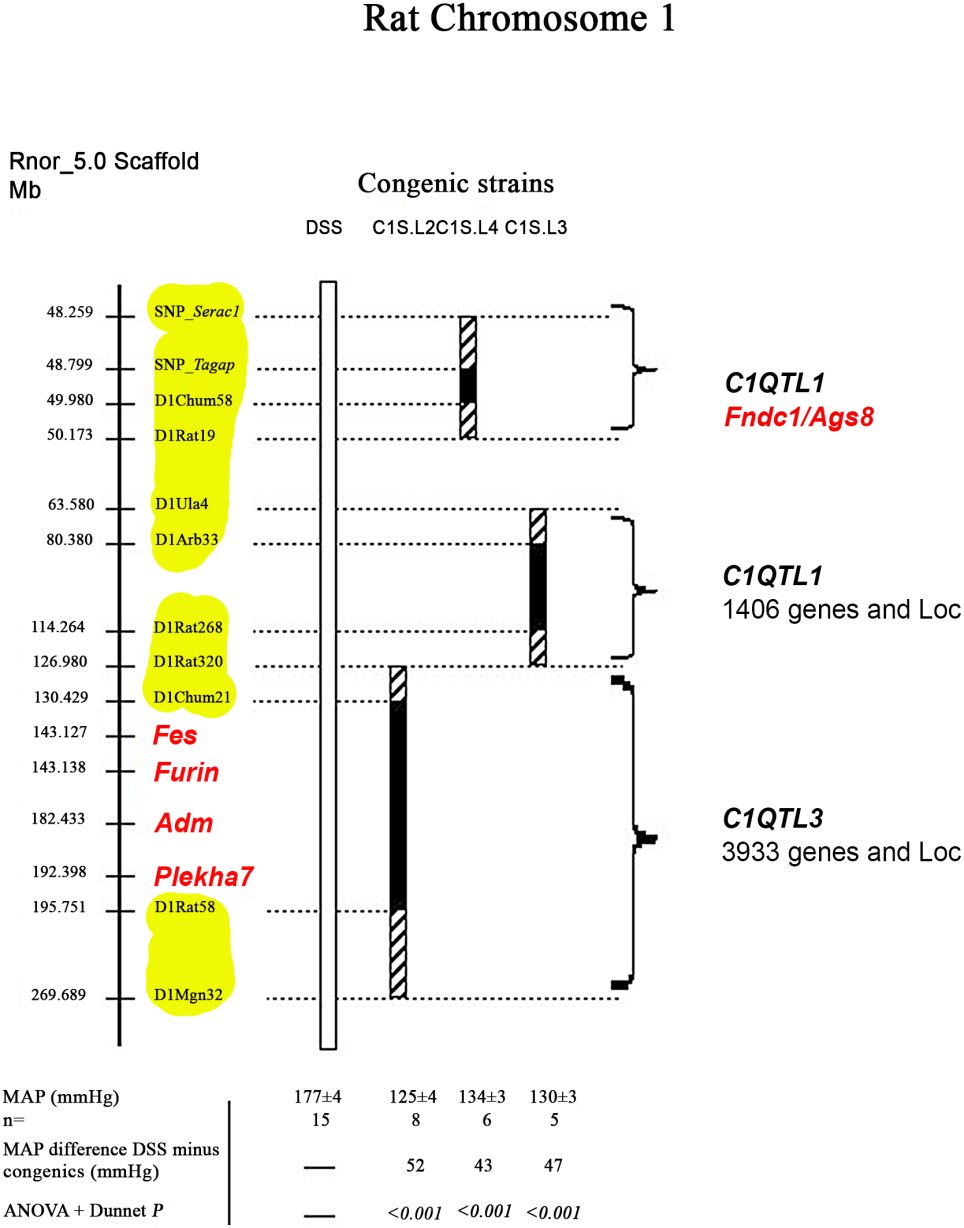
Defining BP QTLs on DSS Chr 1 by congenic strains. A solid bar under congenic strains represents the DSS fragment (a white bar) that has been replaced by that of Lewis (S.L). Striped bars on ends of the solid bars denote the ambiguity of crossover breakpoints between markers. The map is not drawn to scale. Mean arterial pressures (MAPs) for all the strains are averages for the duration of the measurement. Systolic and diastolic arterial pressures are consistent with their MAPs of all the strains (data not shown). *Fibronectin type III domain containing 1* (***Fndc1***)/ *activator of G protein signaling 8* (***Ags8***).

Protocols for handling, keeping and treating animals in our current study have been approved by COMITÉ INSTITUTIONNEL DE PROTECTION DES ANIMAUX DU CRCHUM (CIPA). The congenic approach simulates the ‘knock- in’ principle where a Chr 1 segment of the hypertensive DSS rat has been replaced by that of normotensive Lewis rat, while maintaining the remaining genome as that of DSS [5]. All the congenic strains used in the current study are depicted in Fig 1 and came from our previous work [5]. Their designations are abbreviated to facilitate cross-referencing and presentations in the text and with our previous work.

### Whole genome sequencing of DSS and Lewis rats

We first extracted genomic DNAs from kidneys of DSS and Lewis rats using a QIAamp DNA Mini Kit following the protocol provided by the manufacturer. 5 micrograms of extract DNAs for each rat strain were then sequenced by the Illumina technology at the Mcgill Genome Center (http://gqinnovationcenter.com/index.aspx). S1 Table outlines the methods, procedure, interpretation, sequence calling and data mining along with relative references. The genome sequences became our data base for identifying single-nucleotide polymorphisms (SNPs) differentiating DSS from Lewis (S2 Table). Our results are consistent with those published by other investigators [8].

**Fig 2:**
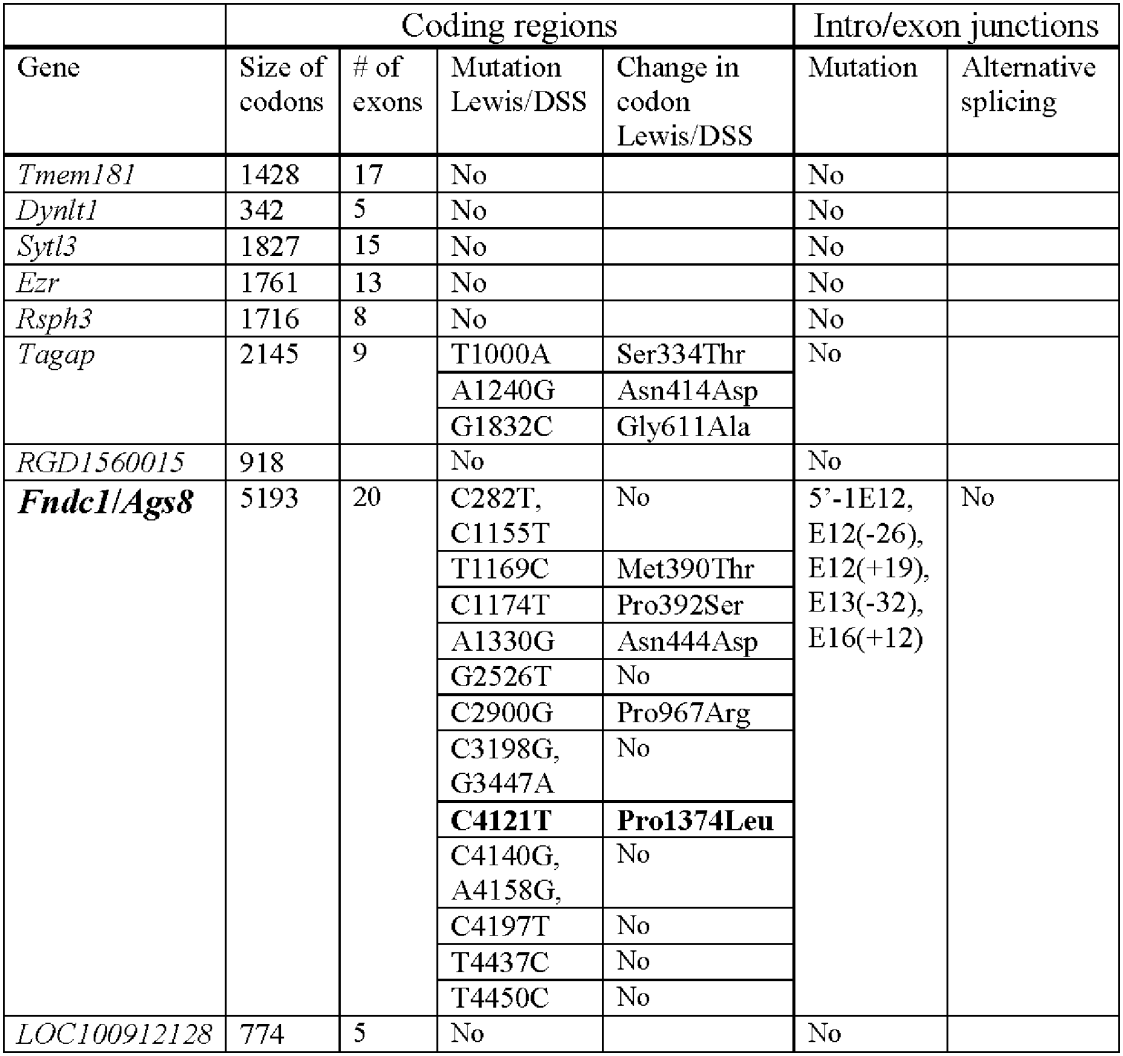
Mutation screening of genes in the *C1QTL1* -containing interval. The position of a mutation enumerates from the ATG start codon of that gene. The amino acid position begins from the first methionine. ***Dynlt1***, *dynein light chain Tctex-type 1*; ***Ezr***, *ezrin*; ***Fndc1/Ags8***, *fibronectin type III domain containing 1*/ *activator of G protein signaling 8* (S3 Table); ***LOC100912128***, *parkin coregulated gene protein-like*; ***RGD1560015***, *similar to glycoprotein*, *synaptic 2*; ***Rsph3***, *radial spoke 3 homolog (Chlamydomonas)*; ***Sytl3***, *synaptotagmin-like 3*; ***Tagap***, *T-cell activation RhoGTPase activating protein* (S4 Table); ***Tmem181***, *transmembrane protein 181*. Pseudogenes are not included. In: intron, Ex: exon, (+) and (-): nucleotide after before a given exon respectively. Raw genome sequence data have been deposited at the European Nucleotide Archive with accesion #s: Accession#s: LT158646, LT158647, LT158648, and LT158649.

Coding regions and intron-exon junctions of all the genes residing in the *C1QTL1*-containing interval (Fig 2) were curated from our genome data base and in consultation with those in the public domain [8]. When a non-synonymous mutation was found, the DNA segment was independently PCR-amplified and re-sequenced including DSS and Lewis rats by Sanger sequencing. Thus, all missense mutations were verified and are homozygous for the Lewis alleles, (i.e. LL) in the C1S.L4 congenic strain that defines *C1QTL1*.

All the genes that carry missense mutations were assessed for expressions by reverse transcriptase (RT) polymerase chain reaction (PCR) in a panel of mRNAs extracted from various organs of DSS and congenic (or Lewis) rats. Copy number variations can also be found.

Quantitative real-time RT PCR (qPCR) was utilized to determine and compare expressions of a gene as reported previously [9]. Data was analyzed by applework5.

## Results

### Overall strategy in identifying candidate genes for a BP QTL

A summary of genome SNPs is presented in S2 Table and illustrates a global distribution of SNPs in known genes between DSS and Lewis genomes. Combining the genome information with a congenic strain defining a BP QTL, *C1QTL1*, we proceeded to identify all the SNPs located in the coding regions and intron-exon junctions of every gene residing in the chromosome interval harboring the QTL.

### Restricting a chromosome interval harboring *C1QTL1*

Congenic strains are powerful in that, first, they isolate the genes functionally impacting on BP based on a cause-effect relationship from a myriad of genes existing in the entire genome [6]. Second, they are capable of separating multiple closely-linked BP QTLs. Genomic SNPs enabled us to curtail the size of chromosome segments harboring BP QTLs on Chr 1 by diminishing the ambiguous regions on both sides of congenic strains as we reported previously [5]. Among the 3 BP QTLs delineated on Chr. 1 (Fig 1), the region harboring *C1QTL1* is narrowed to a segment carrying 9 genes. The number of genes in the other QTL-residing fragments is over 1400 for limiting candidate genes for a detailed molecular study. Because of it, further genetic studies were not pursued for other QTLs.

### *Fndc1* is a novel candidate gene for *C1QTL1*

(a), the congenic strain, C1S.L4, is different from DSS in the segment of 1.9 megabases (Fig 1). This segment-‘knock in’ strain lowers mean arterial pressure (MAP) by 43 mmHg (*p*<0.001) from that of DSS (Fig 1), indicating that a gene(s) entrapped within and among the 9 genes is responsible for the functionality of *C1QTL1*. This level of narrowing candidate genes is one of a few studies so far reported in the experimental genetics of polygenic hypertension.

All the genes, which exist in the *C1QTL1*-containing interval and carry SNPs, are homozygous in genotype for Lewis rats (i.e. LL) in the congenic strain C1S.L4, and were curated from our DSS and Lewis genome data bases. The genes carrying SNPs in the coding regions and intron-exon variations are summarized in Fig 2.

(b), two genes carry non-synonymous mutations, i.e. *Fndc1* (S3 Table) and *T-cell activation RhoGTPase activating protein* (*Tagap*) (S4 Table). In contrast, all the remaining genes located in the *C1QTL1*-residing interval encode identical proteins.

**Fig 3:**
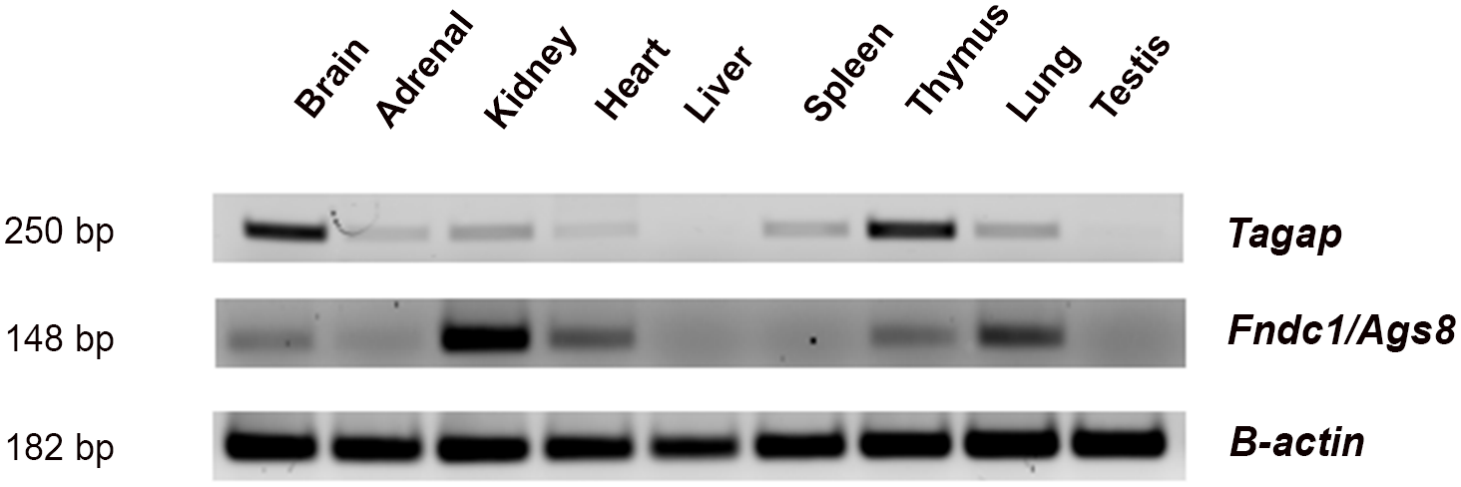
Organ expression pattern of genes assayed by reverse transcriptase polymerase chain reaction (RT-PCR). The organs are from Dahl salt-sensitive (DSS) rats. Numbers to the left indicate the size of the fragment in base pairs. The full gene names are given in the legend in Fig 1 and contain missense mutations. Primers for RT-PCRs of genes are forward 5’ GGGAGAACATTCCGACACAT 3’ and reverse 5’ GCTACCATGCTGACACTGGA 3’ for *Tagap*; forward 5’ TGAAGCTGGCAACCTCATAA 3’ and reverse 5’ TGTGGTCTCAAGTCCATAATCG 3’ for *Fndc1*/*Ags8*; and forward 5’ ACTGCCGCATCCTCTTCCTC 3’ and reverse 5’ CCGCTCGTTGCCAATAGTGA 3’ for *β-actin*. Two primers for each gene are located in 2 different exons to avoid amplifying genomic DNAs contaminated in RNA preparations, since no products were seen when genomic DNAs were amplified (data not shown). Before RT-PCR, all the mRNA samples were treated with RNase-free DNAs to remove possible traces of genomic DNAs. Results shown are from 1 rat of each strain and they have been replicated with multiple rats of the same strain and Lewis rats (data not shown). All rats were males, 11 weeks of age and fed a high salt diet for 6 weeks starting from 5 weeks of age.

No DSS/C1S.L4 alternative splicing was detected for the genes carrying intron-exon boundary variants (Fig 2). When a SNP is found near a splice donor or recipient site, the status of alternative splicing, or a lack of it, is assessed by RT-PCRs of the exons flanking it as follows. While taking into considerations that a gene might be differentially spliced in an organ-specific style, PCR primers located in 1 or 2 exon before and after the implicated exon are used to amplify a panel of cDNA segments from the organs where the gene is expressed (e.g. Fig 3). The resultant products were then analyzed by gel electrophoresis for size polymorphisms. So far, among 9 genes in the *C1QTL1*-residing interval (Fig 2), only *Fndc1*/*Ags8* carries possible splice-site mutations, but they did not influence splicing.

Thus, this mutation screening narrowed the list of probable candidates down to the 2 genes, *Fndc1* and *Tagap*. No copy number variations, short insertions and deletions, structural variants were detected among the genes from the total genome comparisons between DSS and Lewis rats (data not shown).

(c), we analyzed gene expressions on these 2 genes. Fig 3 shows that they are expressed in several organs and are functional genes, not pseudogenes.

(d), we reasoned that for a gene to remain a meaningful candidate for *C1QTL1*, it should carry a missense mutation(s) that would functionally affect the protein product it encodes. We examined the potential functional impact of each missense mutation in the 2 genes (Table 1). Only the Pro1374Leu substitution of *Fndc1* caused by the C4121T mutation is predicted to be damaging (Table 1), and thus may have a functional consequence. In contrast, the mutations in *Tagap* are predicted to be benign or neutral. Thus, *Fndc1* is the best supported candidate gene for *C1QTL1*.

**Table 1:** Functional predictions of amino acid substitutions caused by missense mutations in genes in the *C1QTL1*-residing interval.

	Lewis/DSS	Provean	PolyPhen-2
***Tagap***	Ser334Thr	Neutral	benign
	Asn394Asp	Neutral	benign
	Gly611Ala	Neutral	benign
***Fndc1***	Met390Thr	Neutral	unknown
	Pro392Ser	Neutral	unknown
	Asn444Asp	Neutral	unknown
	Pro967Arg	Neutral	unknown
	**Pro1374Leu**	**Deleterious (-3.681)**	**Possibly damaging**

Polyphen-2 [10] (http://genetics.bwh.harvard.edu/pph2/), and Provean/SIFT [11] (http://sift.jcvi.org/) refer to 2 prediction programs used for assessing a probable consequence of a missense mutation on the function of a protein it encodes. ***Fndc1/Ags8***, *fibronectin type III domain containing 1*/ *activator of G protein signaling 8*; ***Tagap***, *T-cell activation RhoGTPase activating protein*. DSS, Dahl salt-sensitive rats.

(e), *Fndc1* is also known as the activator of G protein signaling 8 (*Ags8*)/the ischemia-inducible regulator of Gβγ subunit [12,13]. The expression of *Ags8*/*Fndc1* is induced specifically in cardiomyocytes in response to ischemia/hypoxia, but not under other cardiac dysfunctions including prolonged tachycardia, Ang II-infused hypertrophy, and heart failure [12]. By implication, the heart seems an important organ where Ags8/Fndc1 acts. Since the pumping action of the heart is a main mechanical force in driving BP [14], we compared the cardiac *Ags8*/*Fndc1* expressions between DSS and congenic strain C1S.L4 (Fig 1). As shown in Table 2, *Ags8*/*Fndc1* is expressed greatly more in C1S.L4 than in DSS, indicating that it is differentially expressed due to the congenic ‘knock-in’.

**Table 2:** Comparison of *fibronectin type III domain containing 1*/ *activator of G protein signaling 8* (*Fndc1*/*Ags8*) expressions by quantitative real time reverse transcriptase polymerase chain reaction (qRT-PCR) in the kidneys and heart.

Organs	Mean R	Ratio of mean R DSS/mean R C1S.L4	*p*
**Kidneys**	1.16x10^-3^ ± 1.41x10^-5^ (C1S.L4)/ 3.66x10^-4^ ± 1.43x10^-4^(DSS)	2.90	0.031
**Heart**	9.39x10^-5^ ± 2.38x10^-5^(C1S.L4)/ 3.30x10^-7^ ± 9.42x10^-9^(DSS)	285	0.014

R is the average value of triplicates from 1 rat and represents the ratio of the expression of the gene target relative to that of the *Gapdh* reference. Mean R is the average value of R from 3 rats of the same strain. C1S.L4 is a congenic strain defining *C1QTL1* in Fig 1. ± refers to SEM. *P*, *t*-test. Primers are located in 2 separate exons and were designed to span at least 1 intron to eliminate the amplification of genomic DNAs contaminated in RNA preparations. The RNA samples were treated with RNase-free DNase before qRT-PCR. Forward primer is 5’- tgaagctggcaacctcataa-‘3; Revrse 5’- tgtggtctcaagtccataatcg-’3. DSS, Dahl salt-sensitive rats.

(f), we noted from the organ expression pattern that *Ags8*/*Fndc1* appears vastly expressed in kidneys (Fig 2), which is consistent with a quantitative organ assessment by other investigators [12]. Since kidneys play a vital role in long-term BP modulations [15], we compared the renal *Ags8*/*Fndc1* expressions (Fig 1). C1S.L4 showed a higher level of *Ags8*/*Fndc1* expression than DSS (Table 2).

**Fig 4:**
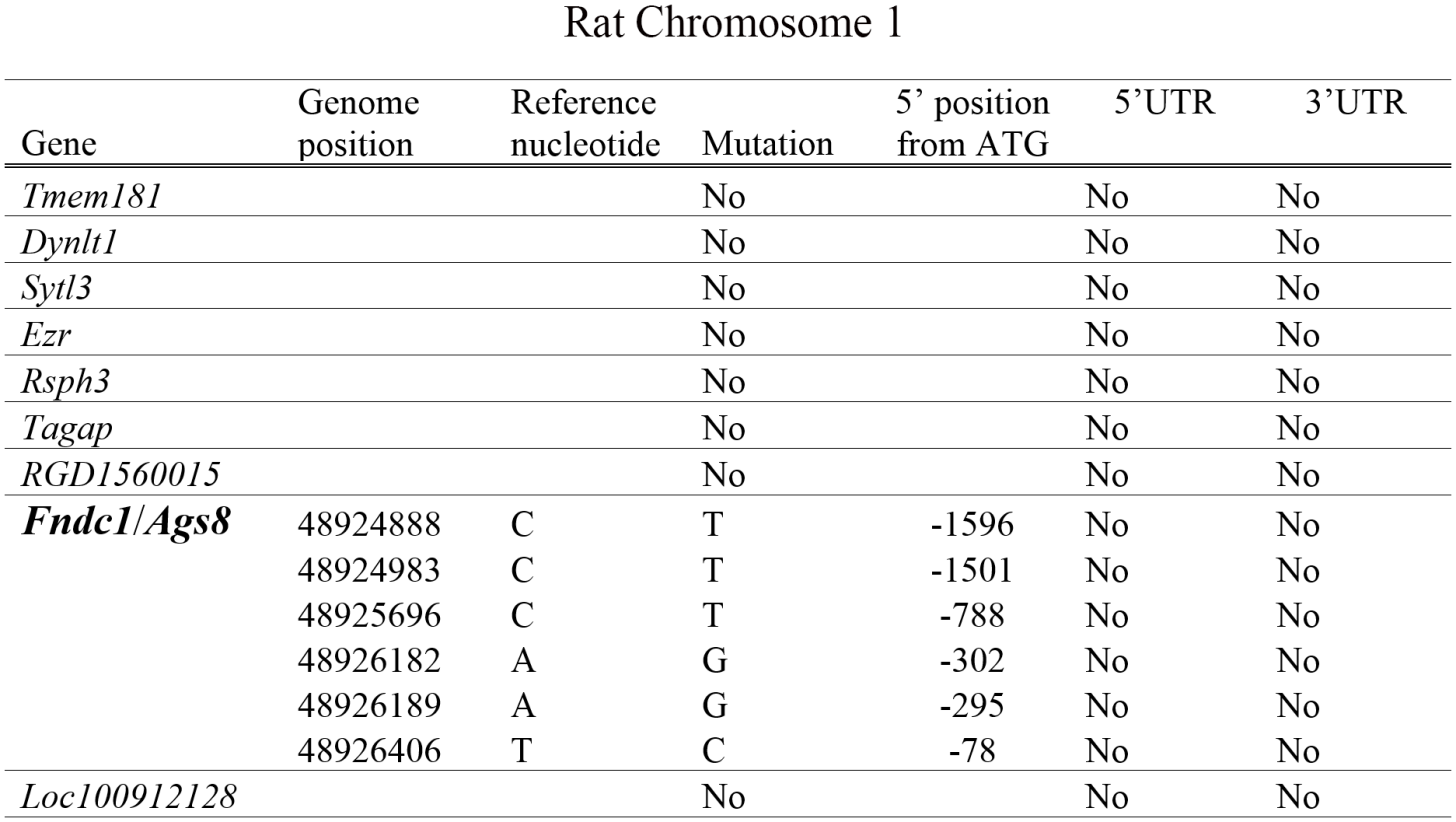
Genome (probable promoter) sequence comparisons up to 2 kilobases from the ATG start codon for the genes in the *C1QTL1*-lodging interval. Gene names are given in the legend for Fig 2. Nucleotides were trolled from our sequence data base of DSS and Lewis genomes (S2 Table). RGD1560015 curated in this region shares a high conserved homology with *Tecr trans-2*,*3-enoyl-CoA reductase* (*Tecr*) on Chr 19, thus cannot be differentiated from it. UTR, untranslated region.

Western blotting using antibodies against Ags8/Fndc1 (Y-12, Santa Cruz) could not detect the protein in the heart and kidneys of DSS and C1S.L4 rats (data not shown), presumably because the protein quantity is below the detectable level in the organs, although a differential expression is seen by a real time RT-PCR after their mRNAs were greatly amplified.

(g), finally, we reasoned that if the *Ags8*/*Fndc1* differential expression is an inherent property of the gene itself, rather than a responder to the action of another gene *in trans* outside the *C1QTL1*-residing segment in the genome, there should be a mutation(s) present in its promoter. Indeed, several mutations are found in its putative promoter (Fig 4, S5 Table) and can potentially account for its differential expression capability.

In contrast, no mutations are found in the 5’ genomic regions ahead of ATG start codons for the remaining genes (Fig 4). This lack of nucleotide variations lessens the chance that any of these genes can be differentially expressed owing to its direct and innate promoter activity, and thus does not support them as possible candidate genes for *C1QTL1*. However, a mutation can not be excluded in other regulatory regions in addition to the promoter that may still have a function impact.

In contrast to the *C1QTL1*-residing segment carrying 9 genes, the chromosome regions containing *C1QTL2* and *C1QTL3* are too large for identifying a single strong candidate gene that either bears structural mutations or exhibits differential expressions. Thus, no additional studies were performed due to this limitation.

## Discussion

The primary finding from the current work is that *fibronectin type III domain containing 1* (*Fndc1*)/*activator of G protein signaling 8* (*Ags8*) is a prohibited candidate gene for *C1QTL1*. The fibronectin type III domain refers to internal repeats found in 2% of animal proteins including extra-cellular matrices, cell-surface receptors, enzymes and muscle proteins [16]. *Ags8*/*Fndc1* was previously shown to be up-regulated in cardiomyocytes when induced by ischemia/hypoxia [12], and highly expressed in kidneys without induction.

From our current work, *Ags8*/*Fndc1* is supported as *C1QTL1* for the following reasons. First, protein-altering mutations, particularly C4121T (Fig 2, Table 1), in *Ags8*/*Fndc1* are associated with a change in blood pressure (Fig 1). Second, *Ags8*/*Fndc1* is differentially expressed greatly in the kidneys and heart (Table 2) supported by numerous promoter mutations (Fig 4, S5 Table).

Gβγ signaling pathways in which Ags8/Fndc1 participate are quite diverse, and some have been linked to hypertension [17]. A *Gβ3* splice variant was associated with increased hypertension in humans [18]. Ags8/Fndc1, a proapoptotic factor, has been shown to mediate the Gβγ-dependent phosphorylation of connexin 43 (Cx43) affiliated with cell permeability and a Cx43 internalization [13]. Interestingly, an endothelium-specific *Cx43* mouse knockout caused hypotension and increased plasma nitric oxide content and, paradoxically an elevated Ang II level [19]. Thus, regular endothelial Cx43 gap junctions may play a role in maintaining the vascular function and a hypertensive state [20]. A *Cx43* knock-in mouse strain by *Cx32* remains normotensive in a 2-kidney and 1-clip model associated with a lower level of renin [21], indicating that Cx43 may mediate hypertension via renin in that model.

In contrast, an inhibition of nitric oxide synthase (Nos), a promoter of hypotension, caused a decrease in aortic Cx43 accompanied by hypertension [22] and correlated with a lower level of phosphorylation of Cx43 in the Nos model. The phosphorylation of Cx43 is a prominent mechanism regulating its function and its cellular trafficking [23].

**Fig 5:**
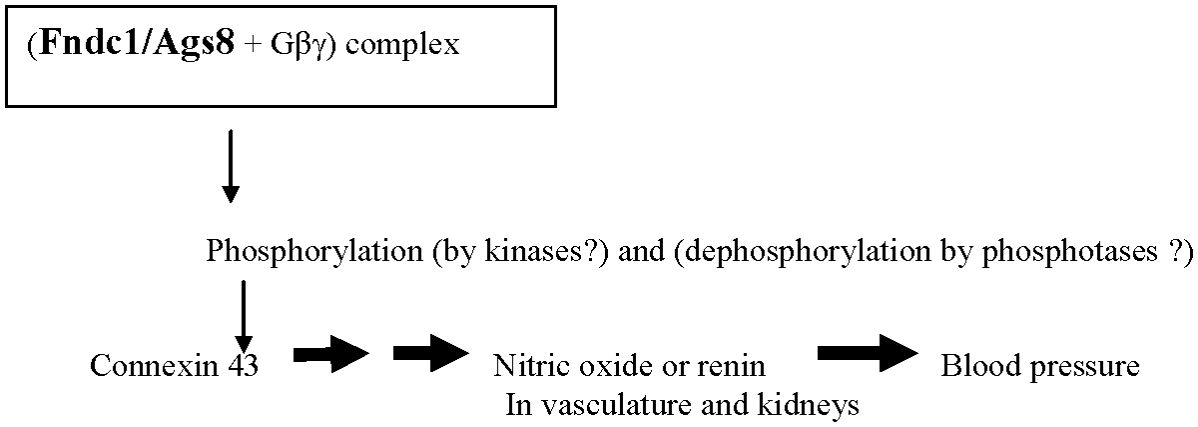
A proposed pathway in which Fndc1/Ags8 participates in potentially contributing to BP modulations. Arrows only indicate a general direction of a pathway and do not enumerate exact steps involved.

The Ags8/Fndc1 involvement in Cx43 phosphorylation may be through activations of kinases by the Ags8/Fndc1-Gβγ pathway [23]. Our genetic work suggests that alterations and a differential production in a positive regulator, Ags8/Fndc1, upstream in a Gβγ signaling pathway may trigger BP changes. As such, *Ags8*/*Fndc1* as *C1QTL1* seems to act ‘indirectly’ on BP far away from the ‘direct’ genes closer to the end-stage BP biology (Fig 5). Interestingly, the gene encoding a new kinase, *alpha kinase 2* (*Alpk2*), was shown to be a BP QTL candidate [9]. *Alpk2* may operate in the same epistatic module/pathway as *Ags8*/*Fndc1*/*C1QTL1* [5]. As such, it is tantalizing to speculate that Alpk2 might phosphorylate Cx43 in connection to the signaling by the Ags8/Fndc1-Gβγ complex.

Since *Ags8*/*Fndc1* is a unique gene that may be required for survival, knocking it out [24] may not be a viable approach in proving its effect on BP. Evidently, no viable mice are available despite the existence of cell lines (http://www.informatics.jax.org/allele/MGI:5000326).

In contrast to the substantial support of *Ags8*/*Fndc1* to be *C1QTL1*, no protein-altering mutations exist in the rat homologues of the human *FURIN/FES*, *ADM*, and *PLEKHA7* (data not shown). Thus, these genes are likely to be markers for the real *C1QTL3* located in their vicinity. Our current work is unique, and yet complements those of other investigators on genetic studies of rat Chr 1, which is rich in BP QTLs [25–27].

### Caveats

Limitations of our current genetic studies are. First, the mechanisms by which the coding mutations in *Ags8*/*Fndc1* change its function are unknown. Second, since Ags8/Fndc1 is pleiotropic even playing a role in response to ischemia/hypoxia, the mechanisms by which Ags8/Fndc1 would regulate the phosphorylation of Cx43 via the Gβγ pathway and lead to the BP modulation are yet to be elucidated (Fig 5). Third, the predicted functional consequences, or their lack of, for missense mutations serve as promising guidelines and need to be verified experimentally.

Fourth, since no gene expression studies were performed for remaining 8 genes, their expressions can still be relevant in a functionally-appropriate organ. Finally, epigenetic variations were not assessed and may exist among the 9 genes.

In conclusion, congenic coupled with molecular studies have identified *Ags8*/*Fndc1* to be the most plausible gene candidate for *C1QTL1*. The combined impact from structural mutations as well as renal and cardiac differential expressions supports a possible functional relevance of *Ags8*/*Fndc1* as *C1QTL1*. *Fndc1*/*Ags8* appears the strongest, not necessarily the only possible, candidate. In other words, *Fndc1*/*Ags8* definitely can not be excluded, whereas the remaining 8 genes may or may not be excluded. They are currently not supported as *C1QTL1* for a lack of genetic evidence except for *Fndc1*/*Ags8*.

*Ags8*/*Fndc1* is known to act distantly upstream in a cascade of signaling mechanisms in modifying CX43 and thus implicates the principle that a QTL might take part in a regulatory hierarchy in a pathway that ‘indirectly’, but eventually results in a BP control [5,28]. Since *Ags8*/*Fndc1* may involve in a pathway with other QTLs [5], revealing its role may pave the way for unfolding the other QTLs in the (Gβγ signaling-CX43 phophorylation-BP) pathway. This conceptual awareness may help unravel the functional roles of certain human QTLs whose rat homologues operate in the same pathway, although no human homologue of *Ags8*/*Fndc1*/*C1QTL1 per se* has been detected [1].

## Supporting Information

S1 Table Total genome sequencing and identification of single nucleotide polymorphisms (SNPs): DNA Sequencing Pipeline Genome Quebec and McGill Innovation Center October 2013. (PDF)

S2 Table Selective global evaluation of genome single nucleotide polymorphisms (SNPs) comparing DSS and Lewis rats. (PDF)

S3 Table Coding sequence alignment of *fibronectin type III domain containing 1* (*Fndc1*)/activator of G protein signaling 8 (*Ags8*) between Dahl salt-sensitive (DSS) and Lewis rats. (PDF)

S4 Table Coding sequence alignment of *T-cell activation RhoGTPase activating protein* (*Tagap*) between Dahl salt-sensitive (DSS) and Lewis rats. (PDF)

S5 Table Sequence alignment of putative *Fndc1/Ags8* promoter between the mouse and Rat. (PDF)
