# Supplementary material for: Alterations in Fibronectin Type III Domain Containing 1 Protein Gene Are Associated with Hypertension
Source: PLoS One. 2016 Apr 11;11(4):e0151399. doi: 10.1371/journal.pone.0151399 (PMC4827815; doi:10.1371/journal.pone.0151399)
Supplement: S1 Table — (PDF) [file pone.0151399.s001.pdf]

S1 Table. Total genome sequencing and identification of single nucleotide polymorphisms (SNPs): DNA Sequencing Pipeline Genome Quebec and McGill Innovation Center October 2013 (bioinformatics.genome@mail.mcgill.ca)

Program	Reference
**Genome Analysis Toolkit**	http://www.broadinstitute.org/gsa/wiki/index.php/The_Genome_Analysis_Toolkit
**BWA**	http://bio-bwa.sourceforge.net/
**igvtools**	http://www.broadinstitute.org/igv/igvtools
**samtools**	http://samtools.sourceforge.net/
**picard**	http://picard.sourceforge.net/
**snpSift/snpEff**	http://snpeff.sourceforge.net/SnpSift.html
**Trimmomatic**	http://www.usadellab.org/cms/index.php?page=trimmomatic

Deliverables: The following files are delivered to clients: (1) A sample statistics file containing the metrics enumerated in Step 6. (2) A .csv file containing all the variants found in at least one sample, often classified by chromosome because of large file sizes. (3) A .csv file containing all the high impact coding variants found in at least one sample, based on the Step 9 annotations.

Sequencing Reads: Around 50 to 150 million 100 b.p. paired-end reads from the Illumina HiSeq 2000 sequencer. Base quality is encoded in phred 33.

Pipeline steps: The pipeline is executed on Compute Canada clusters via unix bash commands, perl scripts and open source software.

Step 1: Read trimming and clipping of adapters: Reads are trimmed from the 3' end to have a phred score of at least 30. Illumina sequencing adapters are removed from the reads, and all reads are required to have a length of at least 32 b.p. Trimming and clipping are done with the Trimmomatic software [1].

Step 2: Aligning the reads to the genome reference: The filtered reads are aligned to the reference genome. The alignment is done per lane of sequencing, and then merged for a complete **B**inary **A**lignment **M**ap file (.bam). The alignment software used is bwa [2], and the merging is done with the picard software [3].

Step 3: Realigning insertions and deletions (INDELs): Insertion and deletion realignment is performed on regions where multiple base mismatches are preferred over indels by the aligner since it can appear to be less costly by the algorithm. Such regions will introduce false positive variant calls which may be filtered out by realigning those regions properly. Realignment is done with the GATK software [4].

Step 4: Fixing the read mates: Once local regions are realigned, the read mate coordinates of the aligned reads need to be recalculated since the reads are realigned at positions that differ from their

original alignment. Fixing the read mate positions is done with picard software [3].

Step 5: Marking duplicates: Aligned reads are duplicates if they have the same 5' alignment positions (for both mates in the case of paired-end reads). All but the best pair (based on alignment score) will be marked as a duplicate in the .bam file. Marking duplicates is done with picard software [3].

Step 6: Compute metrics and generating coverage track: Multiple metrics are computed at this stage and given in the statistics file:

- Number of raw reads
- Number of filtered reads (after Step 1)
- Number of aligned reads (after Step 2)
- Number of duplicate reads (after Step 5)
- Duplicate rate (number of duplicate reads / number of raw reads. Good run max of 25%)
- Median, mean and standard deviation of insert sizes of reads after alignment
- Mean coverage over exons (mean number of reads per base position)
- Percentage of bases covered at X reads (%_bases_above_50 means the % of exons bases which have at least 50 reads. A good run is typically around 50%)

A TDF (.tdf) coverage track is also generated at this step for easy visualization of coverage in the IGV browser [5].

Step 7: Variant calling: Variants (SNPs and INDELs) are called using samtools mpileup and bcftools varfilter [6]. The following options are given to mpileup to filter for low quality variants which could introduce false positive calls: -L 1000 -E -q 1 -u -D -S, where:

-L INT max per-sample depth for INDEL calling [250]
-E extended BAQ for higher sensitivity but lower specificity
-q INT skip alignments with mapQ smaller than INT [0]
-u generate uncompress BCF output
-D output per-sample DP in BCF (require -g/-u)
-S output per-sample strand bias P-value in BCF (require -g/-u)

The output of mpileup is then fed to varfilter, which does an additional filtering of the variants and transforms the output into the VCF (.vcf) format. The arguments used are: -d 2 -D 1200 -Q 15 -1 0.0, where:

-d INT minimum read depth [2]
-D INTmaximum read depth [10000000]
-Q INTminimum RMS mapping quality for SNPs [10]
-1 FLOAT min P-value for strand bias (given PV4) [0.0001]

The final .vcf files are filtered for long 'N' INDELs which are sometimes introduced and causing excessive memory usage by downstream tools.
