# Supplementary material for: Alterations in Fibronectin Type III Domain Containing 1 Protein Gene Are Associated with Hypertension
Source: PLoS One. 2016 Apr 11;11(4):e0151399. doi: 10.1371/journal.pone.0151399 (PMC4827815; doi:10.1371/journal.pone.0151399)
Supplement: S2 Table — (PDF) [file pone.0151399.s002.pdf]

S2 Table. Selective global evaluation of genome single nucleotide polymorphisms (SNPs) comparing DSS and Lewis rats.

Chromosome (Chr)	Gene number	SNP Count	SNPs in genes Count	SNPs in Exon Count	SNPs in intron-exon junctions count
Chr1	2 643	431 290	111 941	3 574	1 588
Chr2	1 115	506 527	98 039	1 886	1 111
Chr3	1 550	263 475	62 965	1 944	986
Chr4	1 185	270 209	78 309	1 505	897
Chr5	1 060	188 601	46 127	1 153	666
Chr6	719	247 020	46 312	1 088	649
Chr7	1 117	245 827	61 742	1 955	1 019
Chr8	1 057	204 003	51 750	1 448	752
Chr9	666	159 279	42 518	655	480
Chr10	1 507	196 741	61 224	2 582	1 367
Chr11	480	141 888	32 930	934	418
Chr12	508	94 706	29 131	815	626
Chr13	586	151 050	38 025	877	573
Chr14	551	124 193	29 395	714	389
Chr15	548	162 540	38 436	797	373
Chr16	492	109 407	30 684	740	478
Chr17	491	121 618	26 337	532	345
Chr18	414	134 343	36 156	1 092	387
Chr19	454	74 450	20 745	637	432
Chr20	592	112 869	32 476	2 139	936

chrX	595	122 396	14 593	490	241
Total	18 330	4 062 432	989 835	27 557	14 713

Gene number refers to the number of known genes. Intron-exon junctions include before and exon sequences up to 100 base pairs.
