# Supplementary material for: Alterations in Fibronectin Type III Domain Containing 1 Protein Gene Are Associated with Hypertension
Source: PLoS One. 2016 Apr 11;11(4):e0151399. doi: 10.1371/journal.pone.0151399 (PMC4827815; doi:10.1371/journal.pone.0151399)
Supplement: S3 Table — (PDF) [file pone.0151399.s003.pdf]

S3 Table. Coding sequence alignment of *fibronectin type III domain containing 1* (*Fndc1*)/activator of G protein signaling 8 (*Ags8*) between Dahl salt-sensitive (DSS) and Lewis rats

```
                 M  G  L  K  V  T  W  D  P  P  K  D  A  T  S  R  P  V  E  H
DSS             ATGGGCCTGAAGGTCACATGGGACCCACCCAAAGACGCTACCAGTAGACCCGTGGAACAT 60
Lewis           ATGGGCCTGAAGGTCACATGGGACCCACCCAAAGACGCTACCAGTAGACCCGTGGAACAT 60
                ************************************************************

                 Y  N  I  A  Y  G  K  S  L  K  S  L  K  S  I  K  V  N  A  E
DSS             TACAACATTGCCTATGGGAAGTCACTGAAAAGTCTTAAGTCCATCAAGGTGAATGCGGAG 120
Lewis           TACAACATTGCCTATGGGAAGTCACTGAAAAGTCTTAAGTCCATCAAGGTGAATGCGGAG 120
                ************************************************************

                           ↓
                 T  H  S  F  L  I  K  D  V  E  K  E  V  P  N  K  P  L  R  M
DSS             ACACACTCCTTCCTTATTAAAGACGTGGAAAAGGAAGTGCCCAACAAGCCCTTGCGTATG 180
Lewis           ACACACTCCTTCCTTATTAAAGACGTGGAAAAGGAAGTGCCCAACAAGCCCTTGCGTATG 180
                ************************************************************

                 R  V  R  A  S  D  D  R  L  S  V  A  W  K  A  P  R  L  S  G
DSS             CGCGTCCGAGCCTCAGATGACAGGCTGTCTGTTGCATGGAAGGCACCACGTCTGTCTGGA 240
Lewis           CGCGTCCGAGCCTCAGATGACAGGCTGTCTGTTGCATGGAAGGCACCACGTCTGTCTGGA 240
                ************************************************************

                 A  K  S  P  R  R  S  R  G  F  L  L  G  Y  G  E  S  G  R  K
DSS             GCCAAGAGCCCACGGAGATCTCGGGGTTTTCTTCTGGCTATGGGGAAAGTGGCCGGAAG 300
Lewis           GCCAAGAGCCCACGGAGATCTCGGGGTTTTCTTCTGGCTACGGGGAAAGTGGCCGGAAG 300
                **************************************** *******************

                                                                  ↓
                 M  N  Y  V  P  L  T  R  D  E  R  S  H  E  I  K  K  L  A  S
DSS             ATGAATTATGTCCCACTGACCAGAGATGAGAGATCGCATGAAATTAAAAAGCTTGCCTCT 360
Lewis           ATGAATTATGTCCCACTGACCAGAGATGAGAGATCGCATGAAATTAAAAAGCTTGCCTCT 360
                ************************************************************

                 E  S  V  Y  V  V  S  L  Q  S  T  N  S  Q  G  Q  S  Q  P  V
DSS             GAGTCGGTGTATGTGGTCTCCCTGCAGTCCACAAACTCCCAGGGGCAGAGTCAGCCCGTC 420
Lewis           GAGTCGGTGTATGTGGTCTCCCTGCAGTCCACAAACTCCCAGGGGCAGAGTCAGCCCGTC 420
                ************************************************************

                                                 ↓
                 Y  R  A  A  L  T  K  R  K  N  A  E  E  D  E  L  D  V  P  E
DSS             TATAGAGCTGCCCTCACAAAGCGCAAGAATGCAGAAGAGGATGAACTGGATGTACCTGAA 480
Lewis           TATAGAGCTGCCCTCACAAAGCGCAAGAATGCAGAAGAGGATGAACTGGATGTACCTGAA 480
                ************************************************************

                 D  I  S  V  R  V  M  S  S  Q  S  V  L  V  A  W  V  D  P  L
DSS             GACATTAGTGTCCGGGTCATGTCGTCTCAGTCCGTGCTCGTGGCCTGGGTGGATCCTCTT 540
Lewis           GACATTAGTGTCCGGGTCATGTCGTCTCAGTCCGTGCTCGTGGCCTGGGTGGATCCTCTT 540
                ************************************************************

                                               ↓
                 V  E  K  Q  K  R  V  V  A  S  R  Q  Y  T  V  R  Y  R  E  K
DSS             GTGGAAAAACAGAAGAGAGTTGTTGCATCAAGACAGTACACTGTGCGCTACCGTGAGAAG 600
Lewis           GTGGAAAAACAGAAGAGAGTTGTTGCATCAAGACAGTACACTGTGCGCTACCGTGAGAAG 600
                ************************************************************

                 G  E  S  A  R  W  D  Y  K  Q  V  S  N  R  R  A  L  V  D  S
DSS             GGGGAGTCGGCCAGGTGGGATTACAAGCAAGTCTCCAACAGGCGTGCACTGGTGGACAGC 660
Lewis           GGGGAGTCGGCCAGGTGGGATTACAAGCAAGTCTCCAACAGGCGTGCACTGGTGGACAGC 660
                ************************************************************

                 L  I  P  D  T  V  Y  E  F  A  V  R  I  S  Q  G  E  R  D  G
DSS             CTGATCCCAGACACCGTGTATGAATTTGCGGTCCGTATTTCACAAGGAGAGCGAGATGGC 720
Lewis           CTGATCCCAGACACCGTGTATGAATTTGCGGTCCGTATTTCACAAGGAGAGCGAGATGGC 720
                ************************************************************

                                                       ↓

                 K  W  S  A  S  V  F  Q  R  T  P  E  S  A  P  T  T  A  P  E
DSS             AAGTGGAGCGCATCTGTCTTCCAGAGAACGCCGGAATCGGCTCCCACCACAGCGCCTGAG 780
Lewis           AAGTGGAGCGCATCTGTCTTCCAGAGAACGCCGGAATCGGCTCCCACCACAGCGCCTGAG 780
                ************************************************************

                 N  L  R  V  W  P  V  N  G  K  P  T  V  V  T  V  S  W  D  A
DSS             AACCTGAGAGTCTGGCCAGTCAACGGCAAGCCCACGGTTGTCACCGTATCCTGGGATGCA 840
Lewis           AACCTGAGAGTCTGGCCAGTCAACGGCAAGCCCACGGTTGTCACCGTATCCTGGGATGCA 840
                ************************************************************
```

```
                                         ↓
       L  P  E  S  E  G  K  V  K  E  Y  I  L  S  Y  A  P  A  L  K
DSS    TTGCCAGAGTCTGAGGGGAAAGTGAAAGAATACATTCTTTCATATGCCCCGGCTCTCAAA 900
Lewis  TTGCCAGAGTCTGAGGGGAAAGTGAAAGAATACATTCTTTCATATGCCCCGGCTCTCAAA 900
       ************************************************************
       P  F  G  A  K  S  L  T  F  S  G  H  T  T  S  A  L  V  D  G
DSS    CCGTTTGGAGCAAAGTCCCTCACCTTCTCTGGACATACTACTTCTGCCCTCGTGGACGGT 960
Lewis  CCGTTTGGAGCAAAGTCCCTCACCTTCTCTGGACATACTACTTCTGCCCTCGTGGACGGT 960
       ************************************************************
       L  Q  P  G  E  R  Y  L  F  K  I  R  A  T  N  R  R  G  Q  G
DSS    CTGCAGCCTGGGGAACGCTATCTGTTCAAAATCCGGGCCACAAACAGGAGAGGCCAGGGG 1020
Lewis  CTGCAGCCTGGGGAACGCTATCTGTTCAAAATCCGGGCCACAAACAGGAGAGGCCAGGGG 1020
       ************************************************************

                                               ↓
       P  H  S  K  A  F  I  V  A  I  P  T  T  S  S  T  E  A  S  V
DSS    CCACACTCCAAAGCCTTCATTGTCGCTATACCAACAACCAGTTCTACTGAAGCCAGTGTC 1080
Lewis  CCACACTCCAAAGCCTTCATTGTCGCTATACCAACAACCAGTTCTACTGAAGCCAGTGTC 1080
       ************************************************************
       Q  P  N  G  R  D  N  G  K  P  E  K  P  Q  Q  P  S  S  S  A
DSS    CAGCCGAATGGGAGAGACAATGGGAAACCTGAGAAACCACAGCAACCTTCTTCCTCTGCT 1140
Lewis  CAGCCGAATGGGAGAGACAATGGGAAACCTGAGAAACCACAGCAACCTTCTTCCTCTGCT 1140
       ************************************************************

       P  K  V  A  A  S  S  Q  H  T  P  S  A  K  N  V  K  D  A  L
DSS    CCCAAAGTTGCAGCTTCCTCACAACACACGCCCTCAGCCAAAAATGTCAAGGATGCTCTC 1200
Lewis  CCCAAAGTTGCAGCCTCCTCACAACACATGCCCCCAGCCAAAAATGTCAAGGATGCTCTC 1200
       ************** ************* **** **************************
                                    M     P

       S  D  L  K  N  K  I  Q  T  N  G  V  A  P  G  R  T  Q  L  H
DSS    TCGGACTTAAAGAACAAAATCCAGACTAATGGTGTGGCGCCCGGAAGAACCCAGCTTCAC 1260
Lewis  TCGGACTTAAAGAACAAAATCCAGACTAATGGTGTGGCGCCCGGAAGAACCCAGCTTCAC 1260
       ************************************************************
       S  K  V  G  E  L  D  P  Q  S  T  E  V  T  G  E  E  E  L  D
DSS    TCTAAAGTGGGTGAGCTGGATCCTCAGTCCACAGAAGTCACTGGCGAGGAGGAACTGGAT 1320
Lewis  TCTAAAGTGGGTGAGCTGGATCCTCAGTCCACAGAAGTCACTGGCGAGGAGGAACTGGAT 1320
       ************************************************************

       S  L  E  D  P  R  S  S  R  L  E  T  L  N  Q  K  Q  P  L  R
DSS    TCCCTTGAAGATCCTCGTTCATCACGGTTGGAGACCCTAAACCAGAAGCAACCCTTGAGG 1380
Lewis  TCCCTTGAAAATCCTCGTTCATCACGGTTGGAGACCCTAAACCAGAAGCAACCCTTGAGG 1380
       ********* **************************************************
                N

       V  P  S  R  S  G  H  G  A  L  A  P  G  R  T  P  A  R  A  G
DSS    GTACCAAGTAGATCTGGTCATGGGGCTCTGGCTCCTGGCAGGACTCCAGCCAGGGCTGGC 1440
Lewis  GTACCAAGTAGATCTGGTCATGGGGCTCTGGCTCCTGGCAGGACTCCAGCCAGGGCTGGC 1440
       ************************************************************
       L  P  V  L  S  R  K  E  G  M  D  R  R  G  P  S  L  D  P  H
DSS    CTGCCAGTGCTGTCCCGCAAGGAAGGGATGGACAGGCGTGGCCCCTCACTGGACCCCCAT 1500
Lewis  CTGCCAGTGCTGTCCCGCAAGGAAGGGATGGACAGGCGTGGCCCCTCACTGGACCCCCAT 1500
       ************************************************************
       P  H  P  R  V  E  P  S  A  S  S  A  Y  H  Q  L  S  S  T  D
DSS    CCTCATCCTAGGGTTGAACCTTCAGCTTCTTCTGCCTACCACCAGCTCAGCTCTACAGAC 1560
Lewis  CCTCATCCTAGGGTTGAACCTTCAGCTTCTTCTGCCTACCACCAGCTCAGCTCTACAGAC 1560
       ************************************************************
       N  D  S  V  D  R  K  E  D  D  Q  A  G  S  P  D  P  K  A  A
DSS    AATGATTCTGTGGACCGAAAGGAGGATGATCAAGCAGGATCCCCTGACCCTAAAGCTGCC 1620
Lewis  AATGATTCTGTGGACCGAAAGGAGGATGATCAAGCAGGATCCCCTGACCCTAAAGCTGCC 1620
       ************************************************************
       S  S  G  S  S  P  K  N  P  G  R  S  R  P  T  S  A  P  S  R
DSS    TCTTCCGGGTCATCTCCCAAGAATCCAGGCAGGTCCAGGCCAACCTCTGCCCCTAGCCGC 1680
Lewis  TCTTCCGGGTCATCTCCCAAGAATCCAGGCAGGTCCAGGCCAACCTCTGCCCCTAGCCGC 1680
       ************************************************************
       H  A  A  S  N  M  L  R  D  K  S  R  V  H  P  G  T  K  A  A
DSS    CATGCGGCTTCTAACATGCTCAGAGACAAAAGCCGGGTACACCCAGGTACAAAGGCAGCA 1740
Lewis  CATGCGGCTTCTAACATGCTCAGAGACAAAAGCCGGGTACACCCAGGTACAAAGGCAGCA 1740
       ************************************************************
```

```
                  S  S  S  T  S  R  Q  S  H  S  S  T  S  E  E  D  S  S  A  Q
DSS              TCATCATCTACGTCGAGGCAGTCTCATTCTTCCACCAGTGAGGAAGACTCCAGTGCCCAA 1800
Lewis            TCATCATCTACGTCGAGGCAGTCTCATTCTTCCACCAGTGAGGAAGACTCCAGTGCCCAA 1800
                 ************************************************************
                  P  S  R  H  F  P  L  H  R  G  S  S  T  S  P  L  S  R  G  W
DSS              CCCTCGAGACATTTCCCACTGCATAGAGGATCTTCTACATCCCCGCTTTCTAGGGGCTGG 1860
Lewis            CCCTCGAGACATTTCCCACTGCATAGAGGATCTTCTACATCCCCGCTTTCTAGGGGCTGG 1860
                 ************************************************************
                  K  D  R  Q  D  T  H  A  S  S  S  H  T  T  S  R  T  A  S  S
DSS              AAGGACCGCCAGGATACCCATGCCTCTAGTTCCCATACGACTTCCCGGACCGCCAGCTCT 1920
Lewis            AAGGACCGCCAGGATACCCATGCCTCTAGTTCCCATACGACTTCCCGGACCGCCAGCTCT 1920
                 ************************************************************
                  S  H  P  S  A  L  T  E  G  S  E  E  E  D  G  G  A  D  S  D
DSS              TCTCATCCCTCTGCCCTGACAGAGGGCTCTGAGGAAGAGGATGGCGGCGCTGATAGTGAC 1980
Lewis            TCTCATCCCTCTGCCCTGACAGAGGGCTCTGAGGAAGAGGATGGCGGCGCTGATAGTGAC 1980
                 ************************************************************
                  R  A  A  E  D  T  I  R  R  A  E  A  T  A  Q  I  Q  Q  T  R
DSS              AGAGCTGCAGAAGACACCATCAGGCGGGCTGAGGCCACTGCTCAGATCCAACAGACCCGG 2040
Lewis            AGAGCTGCAGAAGACACCATCAGGCGGGCTGAGGCCACTGCTCAGATCCAACAGACCCGG 2040
                 ************************************************************
                  P  G  L  G  H  F  S  L  I  R  N  K  P  F  T  P  H  S  R  N
DSS              CCTGGCTTGGGTCACTTTAGTTTGATACGGAATAAACCCTTCACTCCTCACAGCAGAAAT 2100
Lewis            CCTGGCTTGGGTCACTTTAGTTTGATACGGAATAAACCCTTCACTCCTCACAGCAGAAAT 2100
                 ************************************************************
                  P  N  R  F  P  R  L  R  G  P  R  L  Q  P  S  V  S  P  Q  S
DSS              CCAAACAGATTCCCCAGGCTCCGTGGACCAAGGCTTCAGCCTTCTGTGTCTCCTCAGTCT 2160
Lewis            CCAAACAGATTCCCCAGGCTCCGTGGACCAAGGCTTCAGCCTTCTGTGTCTCCTCAGTCT 2160
                 ************************************************************
                  T  S  A  S  K  V  L  T  R  S  P  S  L  P  A  S  H  T  R  P
DSS              ACCTCAGCCTCGAAAGTCCTCACCAGGTCTCCTTCGCTGCCAGCATCCCACACTAGGCCT 2220
Lewis            ACCTCAGCCTCGAAAGTCCTCACCAGGTCTCCTTCGCTGCCAGCATCCCACACTAGGCCT 2220
                 ************************************************************
                  G  S  D  V  Y  G  D  G  E  D  E  E  P  L  P  A  T  V  I  N
DSS              GGCTCTGATGTCTATGGAGATGGTGAGGATGAAGAGCCTCTTCCAGCCACTGTGATCAAT 2280
Lewis            GGCTCTGATGTCTATGGAGATGGTGAGGATGAAGAGCCTCTTCCAGCCACTGTGATCAAT 2280
                 ************************************************************
                  D  R  T  P  S  Y  P  R  H  P  I  S  G  S  S  D  T  L  R  R
DSS              GACCGTACACCTTCCTATCCCAGGCACCCAATCTCTGGCAGCTCAGACACTCTGAGAAGA 2340
Lewis            GACCGTACACCTTCCTATCCCAGGCACCCAATCTCTGGCAGCTCAGACACTCTGAGAAGA 2340
                 ************************************************************
                  G  P  Q  R  G  A  S  L  Y  R  K  E  P  I  P  E  N  S  K  A
DSS              GGTCCCCAGAGAGGGGCCAGCTTGTACCGGAAGGAGCCCATCCCAGAAAACTCCAAAGCT 2400
Lewis            GGTCCCCAGAGAGGGGCCAGCTTGTACCGGAAGGAGCCCATCCCAGAAAACTCCAAAGCT 2400
                 ************************************************************
                  A  G  A  D  V  P  P  G  G  R  S  P  L  S  S  K  A  Q  G  F
DSS              GCTGGAGCGGATGTACCTCCTGGGGGCAGATCCCCTCTGTCCTCCAAGGCTCAGGGCTTT 2460
Lewis            GCTGGAGCGGATGTACCTCCTGGGGGCAGATCCCCTCTGTCCTCCAAGGCTCAGGGCTTT 2460
                 ************************************************************
                  Q  Q  S  T  T  D  E  G  A  P  Q  T  S  P  A  S  T  S  R  Q
DSS              CAACAGAGCACCACCGATGAAGGTGCTCCTCAAACATCCCCAGCATCCACCAGCCGCCAG 2520
Lewis            CAACAGAGCACCACCGATGAAGGTGCTCCTCAAACATCCCCAGCATCCACCAGCCGCCAG 2520
                 ************************************************************
                  P  S  P  A  R  P  P  A  S  R  S  Q  P  S  P  G  S  T  V  P
DSS              CCGTCTCCTGCTAGACCTCCAGCATCAAGATCACAGCCCTCCCCCGGGTCCACTGTTCCC 2580
Lewis            CCGTCGCCTGCTAGACCTCCAGCATCAAGATCACAGCCCTCCCCCGGGTCCACTGTTCCC 2580
                 ***** ******************************************************
                  R  R  M  T  P  D  R  S  S  E  L  S  S  S  Q  S  K  D  R  S
DSS              AGAAGAATGACACCCGATCGCAGCTCAGAACTCTCTAGTTCTCAGAGCAAGGATCGGTCA 2640
Lewis            AGAAGAATGACACCCGATCGCAGCTCAGAACTCTCTAGTTCTCAGAGCAAGGATCGGTCA 2640
                 ************************************************************
                  L  S  Q  P  K  L  S  V  A  H  A  G  H  D  H  P  H  T  A  N
DSS              CTTTCCCAGCCCAAGCTGTCCGTCGCTCACGCAGGGCATGACCACCCTCACACTGCAAAC 2700
Lewis            CTTTCCCAGCCCAAGCTGTCCGTCGCTCACGCAGGGCATGACCACCCTCACACTGCAAAC 2700
                 ************************************************************
                  S  R  G  V  L  P  S  A  P  Q  N  Q  N  E  G  A  Q  S  T  Y
DSS              TCCCGTGGGGTGCTCCCCTCAGCTCCCCAGAATCAGAATGAGGGTGCCCAGAGCACCTAC 2760
Lewis            TCCCGTGGGGTGCTCCCCTCAGCTCCCCAGAATCAGAATGAGGGTGCCCAGAGCACCTAC 2760
                 ************************************************************
```

```
            E  D  N  S  T  E  I  E  G  P  D  S  R  T  P  T  H  S  A  R
DSS        GAGGACAACAGCACTGAAATTGAGGGTCCAGACTCCCGGACTCCCACTCATTCAGCTCGA 2820
Lewis      GAGGACAACAGCACTGAAATTGAGGGTCCAGACTCCCGGACTCCCACTCATTCAGCTCGA 2820
           ************************************************************

            A  K  D  T  T  P  P  I  L  K  P  R  Q  V  G  S  Q  S  W  S
DSS        GCCAAGGATACCACTCCACCCATTCTCAAACCTCGGCAGGTGGGCTCCCAGTCATGGAGC 2880
Lewis      GCCAAGGATACCACTCCACCCATTCTCAAACCTCGGCAGGTGGGCTCCCAGTCATGGAGC 2880
           ************************************************************

            S  D  N  R  P  Q  R  S  Q  A  G  A  S  E  R  P  I  R  P  G
DSS        AGTGACAACAGACCTCAGCGCAGCCAGGCAGGAGCCTCTGAAAGACCCATCCGGCCTGGC 2940
Lewis      AGTGACAACAGACCTCAGCCCAGCCAGGCAGGAGCCTCTGAAAGACCCATCCGGCCTGGC 2940
           ******************* ****************************************
                              P

            S  T  H  P  R  A  Q  V  P  G  R  A  G  V  Q  A  T  S  V  K
DSS        AGCACCCACCCACGGGCCCAGGTTCCTGGCAGGGCTGGGGTCCAGGCCACGTCAGTGAAG 3000
Lewis      AGCACCCACCCACGGGCCCAGGTTCCTGGCAGGGCTGGGGTCCAGGCCACGTCAGTGAAG 3000
           ************************************************************

            K  V  S  P  S  K  R  P  L  P  L  E  S  Q  Q  S  V  F  A  E
DSS        AAGGTCTCACCTTCCAAGCGGCCCCTGCCACTCGAATCTCAGCAATCGGTCTTCGCTGAG 3060
Lewis      AAGGTCTCACCTTCCAAGCGGCCCCTGCCACTCGAATCTCAGCAATCGGTCTTCGCTGAG 3060
           ************************************************************

            E  E  E  E  N  E  G  M  L  K  G  K  E  D  S  L  S  T  S  V
DSS        GAGGAAGAGGAAAACGAAGGAATGTTAAAAGGCAAAGAAGATTCTCTGTCTACCTCAGTT 3120
Lewis      GAGGAAGAGGAAAACGAAGGAATGTTAAAAGGCAAAGAAGATTCTCTGTCTACCTCAGTT 3120
           ************************************************************

            K  K  W  P  S  S  S  S  P  R  D  K  Y  A  D  R  N  L  D  K
DSS        AAAAAGTGGCCTTCTTCCTCCTCCCCGCGGGACAAGTACGCAGACAGGAACCTTGACAAA 3180
Lewis      AAAAAGTGGCCTTCTTCCTCCTCCCCGCGGGACAAGTACGCAGACAGGAACCTTGACAAA 3180
           ************************************************************

            D  K  A  A  I  G  L  L  V  Q  E  E  N  T  V  P  G  R  R  P
DSS        GACAAGGCTGCCATTGGGCTCCTTGTACAGGAAGAGAACACCGTCCCCGGAAGGCGACCA 3240
Lewis      GACAAGGCTGCCATTGGCCTCCTTGTACAGGAAGAGAACACCGTCCCCGGAAGGCGACCA 3240
           ***************** ******************************************

            P  G  S  P  A  I  A  S  H  P  S  T  R  H  Q  P  R  N  P  A
DSS        CCAGGCAGCCCCGCAATAGCCTCACACCCATCTACCCGGCACCAGCCTCGAAATCCCGCC 3300
Lewis      CCAGGCAGCCCCGCAATAGCCTCACACCCATCTACCCGGCACCAGCCTCGAAATCCCGCC 3300
           ************************************************************

            T  A  S  P  I  A  N  T  H  S  W  P  R  Y  T  T  R  A  P  S
DSS        ACTGCAAGTCCCATCGCAAACACACACTCCTGGCCAAGGTATACCACCCGGGCCCCTTCC 3360
Lewis      ACTGCAAGTCCCATCGCAAACACACACTCCTGGCCAAGGTATACCACCCGGGCCCCTTCC 3360
           ************************************************************

            S  Y  S  S  T  T  P  M  L  S  L  R  Q  R  M  Q  R  R  F  R
DSS        AGCTACTCCTCCACCACACCGATGCTCTCCTTGCGGCAGCGGATGCAGCGGAGGTTTAGG 3420
Lewis      AGCTACTCCTCCACCACACCGATGCTCTCCTTGCGGCAGCGGATGCAGCGGAGGTTTAGG 3420
           ************************************************************

                                                            ↓
            T  P  V  S  R  Q  P  P  P  P  R  P  V  L  T  P  G  Y  N  G
DSS        ACGCCAGTCTCGCGCCAGCCTCCACCACCCAGACCCGTGCTTACACCAGGTTATAATGGA 3480
Lewis      ACGCCAGTCTCGCGCCAGCCTCCACCGCCCAGACCCGTGCTTACACCAGGTTATAATGGA 3480
           ************************** *********************************

            R  P  N  A  E  E  N  I  P  P  G  S  I  G  K  P  N  G  Q  R
DSS        AGACCAAATGCAGAAGAGAACATACCTCCTGGTAGTATTGGAAAACCAAACGGACAGAGA 3540
Lewis      AGACCAAATGCAGAAGAGAACATACCTCCTGGTAGTATTGGAAAACCAAACGGACAGAGA 3540
           ************************************************************

                                           ↓
            I  I  N  G  P  Q  G  T  K  W  V  V  D  L  D  R  G  L  V  L
DSS        ATTATTAATGGTCCTCAAGGAACAAAGTGGGTTGTAGACCTGGATCGTGGCTTGGTGTTG 3600
Lewis      ATTATTAATGGTCCTCAAGGAACAAAGTGGGTTGTAGACCTGGATCGTGGCTTGGTGTTG 3600
           ************************************************************

            N  A  E  G  R  Y  L  Q  D  S  H  G  N  P  L  R  V  R  L  G
DSS        AATGCAGAAGGGCGGTACCTCCAAGACTCCCACGGCAATCCTCTCCGGGTGAGACTCGGG 3660
Lewis      AATGCAGAAGGGCGGTACCTCCAAGACTCCCACGGCAATCCTCTCCGGGTGAGACTCGGG 3660
           ************************************************************
```

```
                     ↓
             G  D  G  R  T  I  V  D  L  G  G  T  P  M  V  S  P  D  G  L
DSS         GGAGACGGTCGCACCATCGTGGATCTTGGAGGAACCCCTATGGTGAGTCCCGATGGCCTC 3720
Lewis       GGAGACGGTCGCACCATCGTGGATCTTGGAGGAACCCCTATGGTGAGTCCCGATGGCCTC 3720
            ************************************************************

             P  L  F  G  Q  G  R  H  G  K  P  V  A  S  A  Q  D  K  P  I
DSS         CCCCTGTTTGGGCAAGGAAGACATGGCAAACCTGTGGCCAGTGCCCAGGATAAACCCATC 3780
Lewis       CCCCTGTTTGGGCAAGGAAGACATGGCAAACCTGTGGCCAGTGCCCAGGATAAACCCATC 3780
            ************************************************************

             L  S  L  G  G  K  P  L  V  G  L  E  V  V  R  T  T  T  Q  V
DSS         TTGAGTCTTGGAGGGAAGCCCTTAGTGGGCTTGGAAGTGGTCAGAACAACCACCCAAGTT 3840
Lewis       TTGAGTCTTGGAGGGAAGCCCTTAGTGGGCTTGGAAGTGGTCAGAACAACCACCCAAGTT 3840
            ************************************************************

             P  T  T  T  M  P  P  S  T  T  T  T  T  V  P  P  T  T  T  L
DSS         CCTACCACAACCATGCCGCCAAGCACAACGACCACCACTGTGCCGCCCACCACAACCCTG 3900
Lewis       CCTACCACAACCATGCCGCCAAGCACAACGACCACCACTGTGCCGCCCACCACAACCCTG 3900
            ************************************************************

             P  P  T  T  T  T  T  R  R  T  T  T  T  R  R  T  T  T  T  R
DSS         CCGCCCACAACCACCACCACCCGCCGGACCACCACCACCCGCCGGACCACCACCACCAGG 3960
Lewis       CCGCCCACAACCACCACCACCCGCCGGACCACCACCACCCGCCGGACCACCACCACCAGG 3960
            ************************************************************

             R  P  T  T  T  T  R  A  T  R  R  T  T  T  T  T  T  T  P  E
DSS         CGTCCCACAACCACAACCCGAGCTACTCGCAGGACAACTACCACTACCACCACTCCGGAA 4020
Lewis       CGTCCCACAACCACAACCCGAGCTACTCGCAGGACAACTACCACTACCACCACTCCGGAA 4020
            ************************************************************

             P  T  T  P  S  P  T  C  P  P  G  T  L  E  H  R  D  E  A  G
DSS         CCTACCACCCCCTCTCCTACCTGTCCTCCTGGGACCCTGGAACACCGGGATGAAGCTGGC 4080
Lewis       CCTACCACCCCCTCTCCTACCTGTCCTCCTGGGACCCTGGAACACCGGGATGAAGCTGGC 4080
            ************************************************************

                                                              ↓
             N  L  I  M  G  S  N  G  I  P  E  C  Y  L  E  E  D  D  F  S
DSS         AACCTCATAATGGGCTCAAATGGGATCCCAGAGTGCTACCTCGAGGAAGATGACTTCTCG 4140
Lewis       AACCTCATAATGGGCTCAAATGGGATCCCAGAGTGCTACCCCGAGGAAGATGACTTCTCC 4140
            **************************************** ****************** 
                                                    P

             G  L  E  I  D  T  A  L  P  T  E  E  D  Y  V  V  Y  D  D  D
DSS         GGCCTGGAGATAGACACGGCACTACCCACGGAAGAGGATTATGTTGTGTACGATGATGAT 4200
Lewis       GGCCTGGAGATAGACACAGCACTACCCACGGAAGAGGATTATGTTGTGTACGATGACGAT 4200
            ***************** ************************************** ***

             Y  G  L  E  T  T  R  P  P  T  S  T  M  P  S  T  T  A  A  T
DSS         TATGGACTTGAGACCACAAGGCCACCGACCAGCACCATGCCCTCAACCACTGCTGCCACA 4260
Lewis       TATGGACTTGAGACCACAAGGCCACCGACCAGCACCATGCCCTCAACCACTGCTGCCACA 4260
            ************************************************************

             P  K  V  V  P  E  Q  G  T  V  S  S  F  P  E  E  E  F  D  L
DSS         CCGAAGGTCGTCCCAGAGCAGGGCACTGTCAGCTCCTTCCCAGAGGAAGAATTTGACCTT 4320
Lewis       CCGAAGGTCGTCCCAGAGCAGGGCACTGTCAGCTCCTTCCCAGAGGAAGAATTTGACCTT 4320
            ************************************************************

                                  ↓
             A  G  K  R  R  F  V  A  P  Y  V  T  Y  L  S  K  D  P  A  A
DSS         GCCGGAAAGAGGCGGTTTGTTGCTCCTTACGTGACCTACCTCAGTAAAGACCCAGCAGCC 4380
Lewis       GCCGGAAAGAGGCGGTTTGTTGCTCCTTACGTGACCTACCTCAGTAAAGACCCAGCAGCC 4380
            ************************************************************

             P  C  S  L  T  D  A  L  D  H  F  Q  V  E  S  L  D  E  L  I
DSS         CCGTGCTCACTGACTGATGCCCTGGACCACTTCCAAGTGGAAAGCCTGGATGAACTCATT 4440
Lewis       CCGTGCTCACTGACTGATGCCCTGGACCACTTCCAAGTGGAAAGCCTGGATGAACTTATT 4440
            ******************************************************** ***

             P  N  D  L  T  K  N  D  L  P  P  Q  H  A  P  R  N  I  T  V
DSS         CCAAATGACCTGACAAAGAACGATCTCCCTCCTCAGCATGCTCCCCGGAACATTACTGTG 4500
Lewis       CCAAATGACTTGACAAAGAACGATCTCCCTCCTCAGCATGCTCCCCGGAACATTACTGTG 4500
            ********* **************************************************

             V  A  M  E  G  C  H  S  F  V  I  V  D  W  N  K  A  I  P  G
DSS         GTCGCCATGGAAGGCTGCCACTCCTTTGTCATTGTGGACTGGAACAAAGCCATCCCTGGA 4560
Lewis       GTCGCCATGGAAGGCTGCCACTCCTTTGTCATTGTGGACTGGAACAAAGCCATCCCTGGA 4560
            ************************************************************
```

```
                            ↓
                 D  V  V  T  G  Y  L  V  Y  S  A  S  Y  E  D  F  I  R  N  K
DSS             GATGTGGTAACAGGATACCTGGTCTACAGCGCCTCTTATGAGGACTTCATCAGGAATAAA 4620
Lewis           GATGTGGTAACAGGATACCTGGTCTACAGCGCCTCTTATGAGGACTTCATCAGGAATAAA 4620
                ************************************************************

                 W  S  T  Q  T  S  S  V  T  H  L  P  I  E  N  L  K  P  N  T
DSS             TGGTCAACTCAGACCTCGTCAGTGACCCATTTGCCCATTGAGAACCTGAAGCCAAACACA 4680
Lewis           TGGTCAACTCAGACCTCGTCAGTGACCCATTTGCCCATTGAGAACCTGAAGCCAAACACA 4680
                ************************************************************

                 ↓
                 R  Y  Y  F  K  V  Q  A  K  N  P  H  G  Y  G  P  V  S  P  S
DSS             AGGTATTACTTCAAAGTTCAAGCGAAAAACCCTCACGGCTATGGGCCTGTCAGCCCTTCA 4740
Lewis           AGGTATTACTTCAAAGTTCAAGCGAAAAACCCTCACGGCTATGGGCCTGTCAGCCCTTCA 4740
                ************************************************************

                                      ↓                             ↓
                 V  S  F  V  T  E  S  D  N  P  L  L  V  V  R  P  P  G  G  E
DSS             GTCTCATTTGTCACAGAATCAGACAATCCTCTGCTGGTTGTGAGGCCACCAGGTGGTGAG 4800
Lewis           GTCTCATTTGTCACAGAATCAGACAATCCTCTGCTGGTTGTGAGGCCACCAGGTGGTGAG 4800
                ************************************************************

                 P  I  W  I  P  F  A  F  K  H  D  P  G  Y  T  D  C  H  G  R
DSS             CCCATCTGGATCCCGTTTGCTTTCAAGCATGACCCCGGCTACACTGACTGCCATGGTCGG 4860
Lewis           CCCATCTGGATCCCGTTTGCTTTCAAGCATGACCCCGGCTACACTGACTGCCATGGTCGG 4860
                ************************************************************

                 Q  Y  V  K  R  T  W  Y  K  K  F  V  G  V  V  L  C  N  S  L
DSS             CAGTATGTGAAACGGACGTGGTACAAAAAGTTTGTGGGAGTTGTTCTTTGTAATTCTCTA 4920
Lewis           CAGTATGTGAAACGGACGTGGTACAAAAAGTTTGTGGGAGTTGTTCTTTGTAATTCTCTA 4920
                ************************************************************

                                                  ↓
                 R  Y  K  I  Y  L  S  D  N  L  K  D  T  F  Y  S  I  G  D  S
DSS             AGGTACAAGATCTACCTCAGTGACAATCTCAAAGACACATTCTACAGCATTGGGGACAGC 4980
Lewis           AGGTACAAGATCTACCTCAGTGACAATCTCAAAGACACATTCTACAGCATTGGGGACAGC 4980
                ************************************************************

                 W  G  R  G  E  D  H  C  Q  F  V  D  S  H  L  D  G  R  T  G
DSS             TGGGGAAGGGGTGAAGACCATTGTCAGTTTGTGGATTCGCACCTGGATGGAAGAACGGGG 5040
Lewis           TGGGGAAGGGGTGAAGACCATTGTCAGTTTGTGGATTCGCACCTGGATGGAAGAACGGGG 5040
                ************************************************************

                                                     ↓
                 P  Q  S  Y  V  E  A  L  P  T  I  Q  G  Y  Y  R  Q  Y  R  Q
DSS             CCTCAGTCTTATGTAGAAGCCCTCCCCACCATTCAAGGCTACTATCGCCAGTACCGTCAG 5100
Lewis           CCTCAGTCTTATGTAGAAGCCCTCCCCACCATTCAAGGCTACTATCGCCAGTACCGTCAG 5100
                ************************************************************

                 E  P  V  S  F  G  H  I  G  F  G  T  P  Y  Y  Y  V  G  W  Y
DSS             GAGCCCGTCAGCTTTGGACACATTGGATTTGGAACCCCCTATTACTATGTGGGCTGGTAC 5160
Lewis           GAGCCCGTCAGCTTTGGACACATTGGATTTGGAACCCCCTATTACTATGTGGGCTGGTAC 5160
                ************************************************************

                 E  C  G  V  S  I  P  G  K  W  -
DSS             GAGTGTGGAGTCTCCATCCCAGGAAAGTGGTAA 5193
Lewis           GAGTGTGGAGTCTCCATCCCAGGAAAGTGGTAA 5193
                *********************************
```

* indicates nucleotide identity. Amino acid sequence is given on top. Genomic DNAs from DSS and Lewis were first curated from our databases of complete DSS and Lewis genome sequences (Supplement 2). When a mutation is detected (shaded), the segment harbouring it was amplified by PCR from both genomic and cDNAs, and then sequenced for confirmation. The amino acid changes caused by individual mutations are indicated by bold and large lettering. ↓ marks the last nucleotide of each exon.
