# Supplementary material for: Alterations in Fibronectin Type III Domain Containing 1 Protein Gene Are Associated with Hypertension
Source: PLoS One. 2016 Apr 11;11(4):e0151399. doi: 10.1371/journal.pone.0151399 (PMC4827815; doi:10.1371/journal.pone.0151399)
Supplement: S4 Table — (PDF) [file pone.0151399.s004.pdf]

S4 Table. Coding sequence alignment of *T-cell activation RhoGTPase activating protein* (*Tagap*) between Dahl salt-sensitive (DSS) and Lewis rats

```
                                           ↓
                 M  K  L  I  S  S  L  D  G  A  K  T  L  N  A  N  N  M  D  T
DSS             ATGAAGTTGATAAGCAGTCTCGATGGTGCAAAAACGCTTAATGCCAACAACATGGACACA 60
Lewis           ATGAAGTTGATAAGCAGTCTCGATGGTGCAAAAACGCTTAATGCCAACAACATGGACACA 60
                ************************************************************

                                    ↓
                 L  I  E  C  Q  S  E  G  D  I  K  V  L  P  L  L  T  S  C  E
DSS             TTAATTGAATGTCAGTCAGAGGGCGATATCAAGGTGCTTCCATTGCTGACGTCATGTGAG 120
Lewis           TTAATTGAATGTCAGTCAGAGGGCGATATCAAGGTGCTTCCATTGCTGACGTCATGTGAG 120
                ************************************************************

                                           ↓
                 S  E  D  S  I  C  Q  L  I  E  V  K  K  R  K  K  V  L  S  W
DSS             AGTGAAGACAGCATTTGCCAGCTAATTGAAGTTAAGAAGAGAAAGAAAGTGCTGTCCTGG 180
Lewis           AGTGAAGACAGCATTTGCCAGCTAATTGAAGTTAAGAAGAGAAAGAAAGTGCTGTCCTGG 180
                ************************************************************
                 P  S  L  M  R  K  L  S  P  S  P  D  F  S  G  S  L  E  P  E
DSS             CCATCTCTCATGAGAAAGCTCTCTCCTTCACCAGACTTCTCTGGGTCATTGGAACCAGAG 240
Lewis           CCATCTCTCATGAGAAAGCTCTCTCCTTCACCAGACTTCTCTGGGTCATTGGAACCAGAG 240
                ************************************************************
                 L  K  V  S  L  F  D  Q  P  L  S  I  I  C  K  E  N  D  T  L
DSS             CTGAAAGTGTCGCTGTTTGATCAACCCTTGTCAATCATCTGTAAGGAGAATGACACACTC 300
Lewis           CTGAAAGTGTCGCTGTTTGATCAACCCTTGTCAATCATCTGTAAGGAGAATGACACACTC 300
                ************************************************************

                              ↓
                 P  R  P  I  Q  D  I  L  T  I  L  C  L  K  G  P  S  T  E  G
DSS             CCTAGACCCATCCAGGACATCCTCACCATCCTCTGCCTTAAAGGTCCTTCAACTGAAGGA 360
Lewis           CCTAGACCCATCCAGGACATCCTCACCATCCTCTGCCTTAAAGGTCCTTCAACTGAAGGA 360
                ************************************************************
                 I  F  R  K  A  A  S  E  K  A  R  K  E  L  K  E  E  L  N  C
DSS             ATATTCAGGAAAGCAGCCAGCGAGAAAGCCCGCAAGGAGCTGAAGGAGGAGCTTAACTGT 420
Lewis           ATATTCAGGAAAGCAGCCAGCGAGAAAGCCCGCAAGGAGCTGAAGGAGGAGCTTAACTGT 420
                ************************************************************

                                                                        ↓
                 G  G  S  V  N  L  N  Q  L  P  V  H  L  L  A  V  V  F  K  D
DSS             GGGGGCTCTGTGAATCTGAACCAGCTCCCTGTGCACCTCCTGGCTGTGGTCTTCAAGGAC 480
Lewis           GGGGGCTCTGTGAATCTGAACCAGCTCCCTGTGCACCTCCTGGCTGTGGTCTTCAAGGAC 480
                ************************************************************
                 F  L  R  G  I  P  L  K  L  L  S  C  D  L  F  E  D  W  M  G
DSS             TTCCTCCGAGGAATCCCCCTGAAGCTGCTCTCCTGTGACCTCTTTGAGGACTGGATGGGA 540
Lewis           TTCCTCCGAGGAATCCCCCTGAAGCTGCTCTCCTGTGACCTCTTTGAGGACTGGATGGGA 540
                ************************************************************

                                                              ↓
                 A  L  E  K  P  S  E  E  D  R  I  E  A  L  K  Q  V  A  D  R
DSS             GCCCTGGAGAAGCCCAGTGAGGAGGACAGGATCGAGGCCCTGAAGCAGGTTGCTGATCGG 600
Lewis           GCCCTGGAGAAGCCCAGTGAGGAGGACAGGATCGAGGCCCTGAAGCAGGTTGCTGATCGG 600
                ************************************************************
                 L  P  R  P  N  L  L  L  L  R  T  L  V  Y  V  L  H  L  I  S
DSS             CTCCCCCGGCCCAACCTCCTTCTGCTCAGGACCTTAGTCTACGTGCTACACCTTATCAGC 660
Lewis           CTCCCCCGGCCCAACCTCCTTCTGCTCAGGACCTTAGTCTACGTGCTACACCTTATCAGC 660
                ************************************************************
                 K  N  A  E  V  N  K  M  D  S  S  N  L  A  I  C  I  G  P  N
DSS             AAGAACGCCGAGGTCAACAAGATGGACTCCAGCAACCTGGCCATCTGCATCGGACCCAAC 720
Lewis           AAGAACGCCGAGGTCAACAAGATGGACTCCAGCAACCTGGCCATCTGCATCGGACCCAAC 720
                ************************************************************
                 M  L  T  L  K  N  D  Q  S  L  S  F  Q  A  Q  R  D  L  N  N
DSS             ATGCTCACACTGAAGAATGACCAGAGCCTGTCCTTCCAGGCCCAGAGGGACCTGAACAAT 780
Lewis           ATGCTCACACTGAAGAATGACCAGAGCCTGTCCTTCCAGGCCCAGAGGGACCTGAACAAT 780
                ************************************************************

                   ↓
                 K  V  K  I  L  V  E  F  L  I  D  N  C  L  E  I  F  G  E  N
DSS             AAGGTTAAGATCTTGGTGGAATTCCTCATTGACAACTGCCTTGAAATATTTGGGGAGAAC 840
Lewis           AAGGTTAAGATCTTGGTGGAATTCCTCATTGACAACTGCCTTGAAATATTTGGGGAGAAC 840
                ************************************************************
```

```
                                                              ↓
            I  P  T  H  P  R  I  T  S  D  D  S  L  E  H  T  D  S  S  D
DSS         ATTCCGACACATCCCCGCATCACTTCTGATGACTCTCTGGAACACACTGACAGCTCAGAC 900
Lewis       ATTCCGACACATCCCCGCATCACTTCTGATGACTCTCTGGAACACACTGACAGCTCAGAC 900
            ************************************************************

            V  S  T  L  Q  N  D  S  A  Y  D  S  N  D  P  D  V  E  P  A
DSS         GTGTCGACTCTGCAGAACGACTCAGCCTATGACAGCAATGACCCGGATGTAGAGCCCGCG 960
Lewis       GTGTCGACTCTGCAGAACGACTCAGCCTATGACAGCAATGACCCGGATGTAGAGCCCGCG 960
            ************************************************************

            N  G  V  A  S  P  C  R  Q  L  E  G  P  T  T  T  V  A  G  M
DSS         AATGGAGTTGCCTCTCCCTGCAGGCAGTTGGAGGGTCCCACTACCACAGTGGCTGGCATG 1020
Lewis       AATGGAGTTGCCTCTCCCTGCAGGCAGTTGGAGGGTCCCTCTACCACAGTGGCTGGCATG 1020
            *************************************** ********************
                                                   S

            D  T  R  G  P  R  N  T  C  E  S  S  S  E  S  S  V  S  M  V
DSS         GATACCCGGGGGCCCCGGAACACCTGTGAGTCGAGCTCAGAATCCAGTGTCAGCATGGTA 1080
Lewis       GATACCCGGGGGCCCCGGAACACCTGTGAGTCGAGCTCAGAATCCAGTGTCAGCATGGTA 1080
            ************************************************************

            A  R  L  K  S  S  I  G  Q  Q  D  R  R  F  S  E  P  N  M  P
DSS         GCCAGGCTGAAAAGCTCCATTGGCCAGCAAGACAGGCGTTTCTCTGAACCCAACATGCCA 1140
Lewis       GCCAGGCTGAAAAGCTCCATTGGCCAGCAAGACAGGCGTTTCTCTGAACCCAACATGCCA 1140
            ************************************************************

            P  S  R  E  C  F  V  G  P  I  T  K  Q  K  L  T  R  S  E  D
DSS         CCCTCACGAGAGTGCTTCGTGGGCCCGATAACCAAGCAAAAGCTAACGAGGAGCGAGGAC 1200
Lewis       CCCTCACGAGAGTGCTTCGTGGGCCCGATAACCAAGCAAAAGCTAACGAGGAGCGAGGAC 1200
            ************************************************************

            S  F  V  L  P  Q  E  A  S  C  S  E  G  D  E  A  E  D  P  F
DSS         AGCTTCGTTCTGCCCCAGGAAGCCTCCTGTTCTGAAGGCGATGAAGCCGAAGATCCCTTT 1260
Lewis       AGCTTCGTTCTGCCCCAGGAAGCCTCCTGTTCTGAAGGCAATGAAGCCGAAGATCCCTTT 1260
            *************************************** ********************
                                                   N

            T  E  E  V  F  P  A  V  E  G  K  P  M  R  P  V  D  L  K  I
DSS         ACAGAGGAAGTCTTCCCAGCAGTTGAAGGCAAACCCATGAGACCAGTGGATTTGAAGATA 1320
Lewis       ACAGAGGAAGTCTTCCCAGCAGTTGAAGGCAAACCCATGAGACCAGTGGATTTGAAGATA 1320
            ************************************************************

            K  N  L  T  Q  G  L  A  S  P  Q  G  P  A  N  K  A  F  S  S
DSS         AAGAACTTGACCCAAGGTTTAGCATCTCCACAGGGACCTGCAAACAAAGCTTTCTCCAGC 1380
Lewis       AAGAACTTGACCCAAGGTTTAGCATCTCCACAGGGACCTGCAAACAAAGCTTTCTCCAGC 1380
            ************************************************************

            F  S  A  G  E  S  L  D  S  S  P  V  P  S  P  S  C  P  K  R
DSS         TTCTCCGCGGGGGAATCTTTGGACAGCTCACCTGTGCCTTCTCCATCCTGTCCCAAGAGA 1440
Lewis       TTCTCCGCGGGGGAATCTTTGGACAGCTCACCTGTGCCTTCTCCATCCTGTCCCAAGAGA 1440
            ************************************************************

            N  F  F  T  R  H  Q  S  F  T  T  K  T  D  K  T  K  P  Q  R
DSS         AACTTCTTCACCAGACACCAGAGTTTCACCACAAAGACGGACAAGACCAAGCCCCAGAGA 1500
Lewis       AACTTCTTCACCAGACACCAGAGTTTCACCACAAAGACGGACAAGACCAAGCCCCAGAGA 1500
            ************************************************************

            E  I  R  K  H  S  M  S  F  S  F  A  S  H  K  K  V  L  P  R
DSS         GAAATTAGAAAGCACTCCATGTCATTTTCCTTTGCGTCTCACAAGAAAGTGCTGCCCCGG 1560
Lewis       GAAATTAGAAAGCACTCCATGTCATTTTCCTTTGCGTCTCACAAGAAAGTGCTGCCCCGG 1560
            ************************************************************

            T  S  S  I  G  S  E  K  S  K  D  F  S  R  D  Q  L  Q  K  D
DSS         ACCTCCAGCATTGGGTCTGAGAAATCCAAAGACTTCTCTAGAGACCAGCTCCAGAAGGAC 1620
Lewis       ACCTCCAGCATTGGGTCTGAGAAATCCAAAGACTTCTCTAGAGACCAGCTCCAGAAGGAC 1620
            ************************************************************

            L  R  K  E  S  Q  L  A  G  R  I  I  R  E  N  E  S  E  I  Q
DSS         TTGAGGAAAGAGAGCCAGCTTGCCGGCAGAATCATCCGGGAAAATGAGTCCGAAATCCAA 1680
Lewis       TTGAGGAAAGAGAGCCAGCTTGCCGGCAGAATCATCCGGGAAAATGAGTCCGAAATCCAA 1680
            ************************************************************

            S  Q  T  N  L  G  S  S  L  S  G  T  W  A  L  S  V  D  N  T
DSS         AGCCAAACAAATCTGGGCTCCAGCTTGTCTGGAACCTGGGCCCTCTCAGTTGATAACACG 1740
Lewis       AGCCAAACAAATCTGGGCTCCAGCTTGTCTGGAACCTGGGCCCTCTCAGTTGATAACACG 1740
            ************************************************************

            F  Q  F  I  D  V  R  K  P  G  S  P  P  S  Y  E  E  A  I  Y
DSS         TTCCAGTTCATTGATGTGAGGAAGCCAGGAAGCCCACCATCTTATGAAGAGGCCATTTAT 1800
Lewis       TTCCAGTTCATTGATGTGAGGAAGCCAGGAAGCCCACCATCTTATGAAGAGGCCATTTAT 1800
            ************************************************************
```

```
                   Y  H  T  S  G  L  T  A  Y  S  A  Q  T  V  G  S  M  R  A  R
DSS               TACCACACATCAGGACTCACAGCCTACAGTGCCCAGACAGTTGGGAGTATGAGAGCAAGA 1860
Lewis             TACCACACATCAGGACTCACAGCCTACAGTGGCCAGACAGTTGGGAGTATGAGAGCAAGA 1860
                  ******************************* ****************************
                                                 G
                   M  L  K  Q  S  M  A  V  P  P  V  P  S  H  H  E  G  E  L  S
DSS               ATGTTGAAGCAGAGCATGGCGGTGCCCCCTGTGCCTTCTCACCATGAAGGTGAGCTCAGT 1920
Lewis             ATGTTGAAGCAGAGCATGGCGGTGCCCCCTGTGCCTTCTCACCATGAAGGTGAGCTCAGT 1920
                  ************************************************************
                   E  G  I  P  G  G  H  R  S  S  S  V  T  E  H  W  T  Q  S  Q
DSS               GAAGGGATACCTGGTGGACACAGATCGTCTTCCGTGACTGAGCACTGGACACAGAGTCAG 1980
Lewis             GAAGGGATACCTGGTGGACACAGATCGTCTTCCGTGACTGAGCACTGGACACAGAGTCAG 1980
                  ************************************************************
                   T  V  H  V  S  V  E  T  R  G  R  S  E  L  H  R  L  R  T  V
DSS               ACTGTCCATGTCTCTGTAGAAACTCGGGGGAGATCTGAGCTACATCGATTGAGGACAGTG 2040
Lewis             ACTGTCCATGTCTCTGTAGAAACTCGGGGGAGATCTGAGCTACATCGATTGAGGACAGTG 2040
                  ************************************************************
                   S  E  S  V  Q  R  A  K  L  D  Y  L  G  Q  Q  R  S  H  L  V
DSS               TCTGAGTCCGTGCAGAGGGCTAAGCTGGACTACCTTGGGCAGCAACGCAGCCACTTGGTC 2100
Lewis             TCTGAGTCCGTGCAGAGGGCTAAGCTGGACTACCTTGGGCAGCAACGCAGCCACTTGGTC 2100
                  ************************************************************
                   F  E  V  D  Q  L  R  C  A  K  E  S  Y  I  -
DSS               TTTGAGGTTGACCAACTCCGATGTGCTAAAGAATCCTACATTTAG 2145
Lewis             TTTGAGGTTGACCAACTCCGATGTGCTAAAGAATCCTACATTTAG 2145
                  *********************************************
```

* indicates nucleotide identity. Amino acid sequence is given on top. Genomic DNAs from DSS and Lewis were first curated from our databases of complete DSS and Lewis genome sequences (Supplement 2). When a mutation is detected (shaded), the segment harbouring it was amplified by PCR from both genomic and cDNAs, and then sequenced for confirmation. The amino acid changes caused by individual mutations are indicated by bold and large lettering. ↓ marks the last nucleotide of each exon.
