# Supplementary material for: Alterations in Fibronectin Type III Domain Containing 1 Protein Gene Are Associated with Hypertension
Source: PLoS One. 2016 Apr 11;11(4):e0151399. doi: 10.1371/journal.pone.0151399 (PMC4827815; doi:10.1371/journal.pone.0151399)
Supplement: S5 Table — (PDF) [file pone.0151399.s005.pdf]

## S5 Table. Sequence alignment of putative *Fndc1/Ags8* promoter between the mouse and rat

```
DSS             ACAACCCATAGAGTTTGGGGGTGGGGTTGTAGAACCTTTAAGAGGTGGGATCTAATGGGA 60
Lewis           ACAACCCATAGAGTTTGGGGGTGGGGTTGTAGAACCTTTAAGAGGTGGGATCTAATGGGA 60
MPRM12959       ------------------------------------------------------------

DSS             GATTTATGTCATTCGAGTTTAGTCCTTGAAGGGGACAGTGAGATCCATCTTCGCTCTCTT 120
Lewis           GATTTATGTCATTCGAGTTTAGTCCTTGAAGGGGACAGTGAGATCCATCTTCGCTCTCTT 120
MPRM12959       ------------------------------------------------------------

DSS             ACTGCTTCCTCACTGCCATGAGGTGGGTAGCGTCACTATACCCTATGATCCCACGACCAT 180
Lewis           ACTGCTTCCTCACTGCCATGAGGTGGGTAGCGTCACTATACCCTATGATCCCACGACCAT 180
MPRM12959       ------------------------------------------------------------

DSS             GGCTGCCTCCTCACAGAGCCAAGAGCAGCAGTAACAATCAACCATGGCCCAGAGACTTCC 240
Lewis           GGCTGCCTCCTCACAGAGCCAAGAGCAGCAGTAACAATCAACCATGGCCCAGAGACTTCC 240
MPRM12959       ------------------------------------------------------------

DSS             AACACCATGACCAACACCAAGCCCTCCCGTGTCTAAGTTGACTCTCTCAGGTATTTTATA 300
Lewis           AACACCATGACCAACACCAAGCCCTCCCGTGTCTAAGTTGACTCTCTCAGGTATTTTATA 300
MPRM12959       ------------------------------------------------------------

DSS             ATAGGGACAAGAAGCCCACCAGCACAGAGTCACGGCAGGGAAGTAACCGGAATGTTTGCA 360
Lewis           ATAGGGACAAGAAGCCCACCAGCACAGAGTCACGGCAGGGAAGTAACCGGAATGTTTGCA 360
MPRM12959       ------------------------------------------------------------

DSS             GACTGCTCTTCTCTCTAGATGGCAATGAATTGTTTTCTGACCTGAGTGAGTGGGAGAAGC 420
Lewis           GACTGCTCTTCTCTCTAGATGGCAATGAATTGTTTTCTGACCCGAGTGAGTGGGAGAAGC 420
MPRM12959       ------------------------------------------------------------

DSS             AAGCGTAGCTCATTTCTCAGGCCCAGGAAGCATCTGACGTTCTGTCCCTCTGGTTTGCTT 480
Lewis           AAGCGTAGCTCATTTCTCAGGCCCAGGAAGCATCTGACGTTCTGTCCCTCTGGTTTGCTT 480
MPRM12959       ------------------------------------------------------------

DSS             ACCTGTGCCTTCTCCCTTACGGGACTTTGGGAGCCTCACATGTGGAGTTGGGATGGGTCC 540
Lewis           ACCTGTGCCTTCTCCCTCACGGGACTTTGGGAGCCTCACATGTGGAGTTGGGATGGGTCC 540
MPRM12959       ------------------------------------------------------------

DSS             TCTGGGGATTATGGTCAGGGCACATCATGTTTTTGTCCTCAGAATGAACATCCTTCCTCT 600
Lewis           TCTGGGGATTATGGTCAGGGCACATCATGTTTTTGTCCTCAGAATGAACATCCTTCCTCT 600
MPRM12959       ------------------------------------------------------------

DSS             AAGGCATCAAATCATCCTCTTTACGAATGTTGTTGACTCTCCCAACCCGCTTTGGTCCAG 660
Lewis           AAGGCATCAAATCATCCTCTTTACGAATGTTGTTGACTCTCCCAACCCGCTTTGGTCCAG 660
MPRM12959       ------------------------------------------------------------

DSS             AGGCCATGGGGCAATGTTCGGAGGGAGGACGGGTGCTGGATGTTACAATCCTTTGTGTTT 720
Lewis           AGGCCATGGGGCAATGTTCGGAGGGAGGACGGGTGCTGGATGTTACAATCCTTTGTGTTT 720
MPRM12959       ------------------------------------------------------------

DSS             GTTCTGTCCAGATGTTTCATCAGTATCAAAATTCACATGCTAACCCATGTGTGTGTCCTC 780
Lewis           GTTCTGTCCAGATGTTTCATCAGTATCAAAATTCACATGCTAACCCATGTGTGTGTCCTC 780
MPRM12959       -------------------------------------------CCCATGTGTG--TCCTC 15
                                                           **********  *****

DSS             ATGAAAGTGTTGTCTCCCCTTTCTTTCCTCTTTGAGATTGCCAGTGCGTTTTTAAAAATG 840
Lewis           ATGAAAGTGTTGTCTCCCCTTTCTTTCCTCTTTGAGATTGCCAGTGCGTTTTTAAAAATG 840
MPRM12959       ATGAAAGTGTTGTCTTCCCTTTCTTTCCTCTATGAGATTGCAAGTGCGTTTTAAAAAATG 75
                *************** *************** ********* ********** *******

DSS             TTATTAAAATTAAGGTTTTCCTCCCCACCTGTTTAGGTCACTTTTTACTTATTTATTTTT 900
Lewis           TTATTAAAATTAAGGTTTTCCTCCCCACCTGTTTAGGTCACTTTTTACTTATTTATTTTT 900
MPRM12959       TTATTAAAATTAAGGTTTTCCTCCCCACTCACTTAGGTCACTTTTTA---ATTTATTTT- 131
                ****************************    ***************   *********

DSS             CTGAAAGTGCCTCGTGTTACTGAATAGTCAGCCATCTTATCATCCAAATCAACCAAACGT 960
Lewis           CTGAAAGTGCCTCGTGTTACTGAATAGTCAGCCATCTTATCATCCAAATCAACCAAACGT 960
MPRM12959       CTGAAAATGCCTCCTGTTTCTGAATAGTCAGTCACCTTATCGTCGAAATTAACTCAGCAT 191
                ****** ****** **** ************ ** ****** ** **** ***  * * *
```

```
DSS             TTCTTTGGCTGTTGCAATTGCTACGTCAATAGGTAGCGGATGTTGCCACAATTTTGCATT 1020
Lewis           TTCTTTGGCTGTTGCAATTGCTACGTCAATAGGTAGCGGATGTTGCCACAATTTTGCATT 1020
MPRM12959       TTGTCTGGCTGTTGCAATTGCTACATCACTATCCAGCACACATTGCTACAACCTTGCATT 251
                ** * ******************* *** **   ***  *  **** ****  *******

DSS             ACAGTTCCAGTTACGCAGTAAAGTGAGAGGTTTTG-TTAGGTAAAATGTTAAATATTAGC 1079
Lewis           ACAGTTCCAGTTACGCAGTAAAGTGAGAGGTTTTG-TTAGGTAAAATGTTAAATATTAGC 1079
MPRM12959       GCAGTTCCAGCTACTCAGGGAACTGAGAGATTTTGGCTAGGTAAAATGTTAGGCACTAGC 311
                 ********* *** ***  ** ****** *****  *************   * ****

DSS             TTGGTAAATTTGGAAACAAAGAAAAACTTAAGATCTCATAATTGGAGACCTGGTGGTACT 1139
Lewis           TTGGTAAATTTGGAAACAAAGAAAAACTTAAGATCTCATAATTGGAGACCTGGTGGTACT 1139
MPRM12959       TTGGTAAATGTGAAAATGAAGAAAAACGTAAGAGTTCATAATTGAAGATGAGGTGGTACT 371
                ********* ** ***  ******* *****  ***** **** ***   *********

DSS             TTTAAAAAGTTATTTGATTTGTAAAATGTTTTGTTTGCATTTATACAAGTGC-------- 1191
Lewis           TTTAAAAAGTTATTTGATTTGTAAAATGTTTTGTTTGCATTTATACAAGTGC-------- 1191
MPRM12959       TT-AAAAAGTTATTTG------------TTTTGTTTGCATTTATGCAAGTGCACCGTGCA 418
                ** *************            **************** *******

DSS             TCATGCTCATTCGGTACAATGCCCTGGAGGCTGGAAGTGGGTGTTGGATCCTTTGGAACT 1251
Lewis           TCATGCTCATTCGGTACAACGCCCTGGAGGCTGGAAGTGGGTGTTGGATCCTTTGGAACT 1251
MPRM12959       CCATGCGCATGCGGCGCGATGCCCAGGAGGCTGGAAGTG----TTGGCTCCTTTGGAACT 474
                 ***** *** ***  * * **** **************    **** ************

DSS             GGAGCCACAGATGATCTTATCCTGTGGGTGCTGGGAACCTAGCATGGGTGTTCTGCACAA 1311
Lewis           GGAGCCACAGATGATCTTATCCTGTGGGTGCTGGGAACCTAGCATGGGTGTTCTGCACAA 1311
MPRM12959       GGAGCCCTAGATGA---------------------------------------------- 488
                ******  ******

DSS             GCGGTCATTGTTCTTAAGCCCTGAGCCATTGTTCCATTCCCCACAATAATCCTTTTTGAT 1371
Lewis           GCGGTCATTGTTCTTAAGCCCTGAGCCATTGTTCCATTCCCCACAATAATCCTTTTTGAT 1371
MPRM12959       ------------------------------------------------------------

DSS             GATCCACTGTTGTTTTCTCTTTTGGGAAACCTTGGAACAATTTAATGATTAGATGTATAC 1431
Lewis           GATCCACTGTTGTTTTCTCTTTTGGGAAACCTTGGAACAATTTAATGATTAGATGTATAC 1431
MPRM12959       ----------------------------------------------------ATGTGTAC 496
                                                                    **** ***

DSS             AAAAAAGAAACCAGAGTTTTACATCTCCTTCAAGTGTGGTTCAGCCTTTGTGGAAACAAA 1491
Lewis           AAAAAAGAAACCAGAGTTTTACATCTCCTTCAAGTGTGGTTCAGCCTTTGTGGAAACAAA 1491
MPRM12959       -------------------CACGT---------------TTTGGCCTTTGTGGAAATGAA 522
                                    ** *               **  *************  **

DSS             CACTTTGTCCCCTCACTTTGAATATGGCGGTTAGTCCTCCTAACCC--ACTGTAGTGCCT 1549
Lewis           CACTTTGTCCCCTCACTTTGAATATGGCGGTTAGTCCTCCTAACCC--ACTGTAGTGCCT 1549
MPRM12959       CACTTTGTCTCCTCACTTTGAACATGGCAGCTGGTCCTCCCAACCCTCACTGTAA----- 577
                ********* ************ ***** * * ******* *****  ******

DSS             TCCCCTTAGAGAGG-TTTCAAAGTTCGTTCAGCCTCACA----AGACACCAGGGGCACAT 1604
Lewis           TCCCCTTAGAGAGG-TTTCAAAGTTCGTTCAGCCTCACA----AGACACCAGGGGCACAT 1604
MPRM12959       --CCCTTAGAGAGGCTTTTAAAGCTTGTTCAGTTTCACACAGAAGACACCAGGAGCCAAT 635
                  ************ *** **** * ******  *****    ********** **  **

DSS             GTAGTGAACAAATGCTTCTGGAGAGTATGTGAAATAAGTTACTCATTTGTTTTTTCTTGC 1664
Lewis           GTAGTGAACAAATGCTTCTGGAGAGTATGTGAAATAAGTTACTCATTTGTTTTTTCTTGC 1664
MPRM12959       GCAGTGAGCAAATCCTTCTGGGGACTGTGTGAAATAAATTACTCATTTGTTTTT-CTTGC 694
                * ***** ***** ******* ** * ********** **************** *****

DSS             CCTGGCACGTCTGCATAATCAAGGCAAGGGACGCCCAGGGTTTATTTTGAATTTCTTCCC 1724
Lewis           CCTGGCACGTCTGCATAATCAAGGCAAGGGACACCCAGGATTTATTTTGAATTTCTTCCC 1724
MPRM12959       CCTGGCATGTCTGCATAATCAAGGCAAGAAATGCCAAGGGTTGATTTTGACTTTCTTCCC 754
                ******* ********************  *  ** *** ** ******* *********

DSS             GTTCGGTTTAAATTTACCACCCACAGTTATCTAAAATGCAAACCGCATGTGGGATCCGCG 1784
Lewis           GTTCGGTTTAAATTTACCACCCACAGTTATCTAAAATGCAAACCGCATGTGGGATCCGCG 1784
MPRM12959       GTTTGGTTTAAATTTACCACCCACGGTAATCCAAAATACAACCCACACGTGGGATCTGTG 814
                *** ******************** ** *** ***** *** ** ** ******** * *
```

```
DSS             CGTTTGTTTCACTGTCACACCGTGACCGGGTGCAAACATGGCTGCATGCACATCTTTAGG 1844
Lewis           CGTTTGTTTCACTGTCACACCGTGACCGGGTGCAAACATGGCTGCATGCACATCTTTAGG 1844
MPRM12959       TGTTTGTTTCGCTGTCATGCTGTGACCGTGTGCAAACATGGTTGCACGCAAATCTTTAGG 874
                 ********* ******  * ******* ************ **** *** *********

DSS             ATCAAACAGATCCGATGTCATTTACGTTCTTGCTGAGTGCGTCAGGGAACACTGGGAGAA 1904
Lewis           ATCAAACAGATCCGATGTCATTTACGTTCTTGCTGAGTGCGTCAGGGAACACTGGGAGAA 1904
MPRM12959       ATCAGACAGATGGAATGTCATTTACATTCTTGCTGAGAATATCAGAGAACATTCAGAGAA 934
                **** ******   *********** ***********    **** ***** *  *****

DSS             CCATCCTCTCAGCC----ACCGCACAAAGTTCATAGCCGCACTGTTTGTATAGTTGACCA 1960
Lewis           CCATCCTCTCAGCC----ACTGCACAAAGTTCATAGCCGCACTGTTTGTATAGTTGACCA 1960
MPRM12959       GTATTCTCCCAGCCTGCCATTGCATAAAGTTCATAGTTGCGCTGTTTGTATAGTTGACCA 994
                  ** *** *****    *  *** ***********  ** *******************

DSS             CCCACTGAAGCCAAGGCATGTGAAACTGCTGTCCGCTAAC- 2000
Lewis           CCCACTGAAGCCAAGGCATGTGAAACTGCTGTCCGCTAAC- 2000
MPRM12959       CCCACTGAAGCCAAGGCATGTGAAACTGCTGTCCACTAACA 1035
                ********************************** *****
```

MPRM12959 refers to the mouse promoter sequence defined by (http://www.genecopoeia.com/product/search/view_seq_promoter.php?cid=&type=promoter&prod_id=MPRM12959). Fibronectin type III domain containing 1 protein (*Fndc1*)/activator of G protein signaling 8 (*Ags8*); DSS, Dahl salt-sensitive rats. The sequence differences between DSS and Lewis are shaded.
